# Real-time intrafraction motion monitoring in external beam radiotherapy

**DOI:** 10.1088/1361-6560/ab2ba8

**Published:** 2019-08-07

**Authors:** Jenny Bertholet, Antje Knopf, Björn Eiben, Jamie McClelland, Alexander Grimwood, Emma Harris, Martin Menten, Per Poulsen, Doan Trang Nguyen, Paul Keall, Uwe Oelfke

**Affiliations:** 1 Joint Department of Physics, Institute of Cancer Research and Royal Marsden NHS Foundation Trust, London, United Kingdom; 2 Department of Radiation Oncology, University Medical Center Groningen, University of Groningen, The Netherlands; 3 Department of Medical Physics and Biomedical Engineering, Centre for Medical Image Computing, University College London, London, United Kingdom; 4 Department of Oncology, Aarhus University Hospital, Aarhus, Denmark; 5 ACRF Image X Institute, University of Sydney, Sydney, Australia; 6 School of Biomedical Engineering, University of Technology Sydney, Sydney, Australia; 7 Author to whom any correspondence should be addressed.; jenny.bertholet@icr.ac.uk

**Keywords:** motion monitoring, IGRT, MR-guided RT, tumour motion, particle therapy, ultrasound imaging, tracking

## Abstract

Radiotherapy (RT) aims to deliver a spatially conformal dose of radiation to tumours while maximizing the dose sparing to healthy tissues. However, the internal patient anatomy is constantly moving due to respiratory, cardiac, gastrointestinal and urinary activity. The long term goal of the RT community to ‘see what we treat, as we treat’ and to act on this information instantaneously has resulted in rapid technological innovation. Specialized treatment machines, such as robotic or gimbal-steered linear accelerators (linac) with in-room imaging suites, have been developed specifically for real-time treatment adaptation. Additional equipment, such as stereoscopic kilovoltage (kV) imaging, ultrasound transducers and electromagnetic transponders, has been developed for intrafraction motion monitoring on conventional linacs. Magnetic resonance imaging (MRI) has been integrated with cobalt treatment units and more recently with linacs. In addition to hardware innovation, software development has played a substantial role in the development of motion monitoring methods based on respiratory motion surrogates and planar kV or Megavoltage (MV) imaging that is available on standard equipped linacs.

In this paper, we review and compare the different intrafraction motion monitoring methods proposed in the literature and demonstrated in real-time on clinical data as well as their possible future developments. We then discuss general considerations on validation and quality assurance for clinical implementation.

Besides photon RT, particle therapy is increasingly used to treat moving targets. However, transferring motion monitoring technologies from linacs to particle beam lines presents substantial challenges. Lessons learned from the implementation of real-time intrafraction monitoring for photon RT will be used as a basis to discuss the implementation of these methods for particle RT.

## 1. Introduction

Radiation therapy (RT) is a cornerstone of cancer treatment owing to its ability to selectively irradiate tumoural tissues while sparing healthy tissues (Jaffray 2012). However, accurate spatial dose delivery is challenging due to changes in internal anatomy occurring on different time scales. Patient set-up as well as day-to-day changes in anatomy such as weight loss or tumour progression or shrinkage, known as interfraction motion, can be monitored using image-guided radiotherapy (IGRT) prior to treatment delivery. However, intrafractional changes due to bladder filling, peristalsis or tumour drift happen on a shorter time scale of minutes which may require intrafraction monitoring. Even faster motion caused by respiration or cardiac activity occurs which affects treatment accuracy and real-time monitoring of this motion requires a high temporal frequency. Respiration-induced target motion (translation, rotation and deformation) of several centimetres has been observed in liver (Case *et al* 2009, Park *et al* 2012, Worm *et al* 2013, Xu *et al* 2014, Bertholet *et al* 2016), lung (Seppenwoolde *et al* 2002, Kyriakou and McKenzie 2012, Huang *et al* 2015, Schmidt *et al* 2016) and pancreas (Ahn *et al* 2004, Jones *et al* 2015, Campbell *et al* 2017a). Cardiac activity can also have a substantial effect on the position of lung tumours, mediastinal lymph nodes (Seppenwoolde *et al* 2002, Chen *et al* 2014, Schmidt *et al* 2016, Scherman Rydhög *et al* 2017) or liver tumours (Kitamura *et al* 2003, Bertholet *et al* 2016). Erratic motion of the prostate, including rotation, was also reported in several studies (Aubry *et al* 2004, Ghilezan *et al* 2005, Kupelian *et al* 2007, Langen *et al* 2008, Poulsen *et al* 2008b, Ng *et al* 2012, Huang *et al* 2015, Hunt *et al* 2016, Tynan *et al* 2016, Chi *et al* 2017).

Motion of the tumour and the surrounding organs during the delivery of a plan designed on a static anatomy may result in tumour underdosage and over-exposure of healthy tissues. In order to mitigate the detrimental effect of motion on dose delivery, margins are a widely used passive approach aiming at ensuring target coverage despite intrafraction motion either by encompassing the entire path covered by the target during pre-treatment imaging using an internal target volume (ITV), or by using probabilistic margins in a mid-ventilation approach (Stroom and Heijmen 2002, van Herk 2004). However, ITV and mid-ventilation approaches may result in large irradiated volumes leading to high dose delivery to the organs at risk (OAR) (Wolthaus *et al* 2008, Ehrbar *et al* 2016, Kamerling *et al* 2016a) while target coverage is not guaranteed, especially in the presence of tumour drift. Active motion mitigation techniques such as tracking or gating (Keall *et al* 2006) allow for margin reduction while ensuring target coverage but this requires real-time motion monitoring to trigger the beam on/off signal during gating or the tracking feedback loop.

Intrafraction motion monitoring and mitigation are particularly needed for stereotactic body RT (SBRT), where an ablative dose is delivered to the tumour in a few fractions and tight margins are needed to spare the healthy tissues. Because of the high dose delivered per fraction, delivery times are also increased with two main consequences. First, large drifts and changes in breathing patterns are more likely to occur within a fraction. Second, set-up and drift-related errors may no longer be considered random in margins recipes (Stroom and Heijmen 2002, van Herk 2004, Herschtal *et al* 2013) and are likely to have a greater impact on dosimetric errors. SBRT with motion mitigation has shown promising clinical outcome for abdominal tumours in the recent years (Su *et al* 2017b, Henke *et al* 2018) and the high disease control rate observed for SBRT of early stage lung cancer patients (Onishi *et al* 2007) is motivating the introduction of dose escalation and SBRT for locally advanced lung cancer patients where targeting accuracy and margin reduction are key due to the large irradiated volumes (Bainbridge *et al* 2017).

The actually delivered dose, taking motion into account, may be estimated from time-resolved motion monitoring data (Poulsen *et al* 2012b, Kamerling *et al* 2017, Ravkilde *et al* 2018) and would arguably allow to establish more accurate dose-response models than the planned dose (Siochi *et al* 2015, Meijers *et al* 2019).

The interest in the RT community to ‘see what we treat, as we treat’ and adapt treatment instantly has led to the development of numerous real-time motion monitoring and mitigation techniques. Fully integrated systems such as robotic linear accelerators (linac) and gimbal steered linacs with imaging suites were specifically designed to combine motion monitoring with mitigation by dynamic tumour tracking and are now routinely used (Hoogeman *et al* 2009, Depuydt *et al* 2014). Magnetic resonance (MR) imaging was also integrated with treatment machines with two commercial systems (Mutic and Dempsey 2014, Raaymakers *et al* 2017) where gating is applied on the MRIdian (Green *et al* 2018, Tetar *et al* 2018) and multi-leaf collimator (MLC) tracking has been proposed on the Unity (Glitzner *et al* 2018). Add-on systems such as electromagnetic transponders, surface imaging and ultrasound transducers may be interfaced with conventional linacs for automatic gating of the treatment beam (Grimwood *et al* 2018, Worm *et al* 2018). In addition, conventional linacs alone may provide 3D motion monitoring capability (Keall *et al* 2018b) and mitigation via MLC tracking (Keall *et al* 2014b, 2018a, Booth *et al* 2016) or couch tracking (Ehrbar *et al* 2017b) although the latter has not been used clinically to date.

In particle therapy, inline motion and anatomical changes along the beam path may have large dosimetric effects that cannot fully be accounted for by the use of margins (Engelsman *et al* 2013, De Ruysscher *et al* 2015). Particle therapy centres have seen the integration of add-on monitoring equipment and on-board imaging similar to that of conventional linac systems. However, efforts to translate motion monitoring approaches from photon therapy to particle therapy are still challenged by the accuracy requirements of particle therapy and the technical challenges of integrating hardware-focused systems in a particle therapy treatment room.

In this review, we present the different real-time motion monitoring methods used clinically in photon or particle therapy and their possible future developments in section 2. Motion mitigation, active or passive, will not be discussed in depth in this review; instead we refer the reader to the AAPM Task group 76 report (Keall *et al* 2006), the paper by Dieterich *et al* (2008) and, for proton therapy, to the consensus guidelines of the PTCOG thoracic and lymphoma subcommittee (Chang *et al* 2017). In section 3, we discuss the validation of motion monitoring methods at the development or early implementation stage (3.1) and general considerations on quality assurance (QA) in clinical practice. In section 4, the translation of the experience from photon therapy to particle therapy will be discussed. Finally, section 5 concludes this review with a discussion of the presented method and an outlook on the expected evolution of motion monitoring in photon and particle therapy.

## 2. Real-time intrafraction motion monitoring methods

In this review, the term ‘monitoring’ will be used for the measurement (or estimation) of the tumour or OAR position as a function of time while the term ‘tracking’ will be used only to refer to the action of following the tumour with the treatment beam. The tumour or OAR being monitored may not be directly visible but monitored using a surrogate (internal or external). In addition, the position of visible tumours and OARs is generally reduced to the centre of mass of the structure. Therefore in this review, the term ‘target’ refers to the surrogate position or to the centre of mass position for the tumour or OAR being monitored. ‘Real-time monitoring’ refers to the measurement and processing (or estimation) of target position using solely information that is available at the time of interrogation (e.g. image acquisition) with a time delay no longer than 0.5 s for the monitoring of respiratory motion. The time delay may be longer for slow motion such as that of the prostate. ‘Online monitoring’ refers to monitoring performed while the patient is on the treatment table. The International Organisation of Standardization (ISO) 5725-1 (ISO 1994) defines the accuracy of a measure as a combination of the trueness (mean error) and precision (standard deviation, SD, of the error). Accuracy is often defined as the mean error in motion monitoring reports. In this review, we use the term accuracy as intended by ISO 5725-1 and use mean and SD to report trueness and precision.

The different motion monitoring methods discussed in this review are listed in table 1. The corresponding sections are indicated in parenthesis in the first column.

**Table 1.** Overview of the technologies used for real-time motion monitoring.

Technology (section)	Internal/external	Dimensions	Additional ionising radiation	Tissue/tumour/surrogate	Additional equipment to standard linac	Online solution (vendor) if applicable
Infrared (2.1.1)	External	1D	No	Patient surface	No	RPM (Varian) respiratory gating (figure 1(a))
		6 DoF		Fixation devices	Yes	IRLED (Brainlab)
Optical (2.1.2)	External	6 DoF surface	No	Patient surface	Yes	Align RT (vision RT) (figure 1(b))/catalyst (C-RAD)
Spirometry (2.1.3)	External	1D	No	Lung volume changes	Yes	ABC (Elekta) (figure 1(c))
Pressure belt (2.1.3)	External	1D	No	Abdomen perimeter	Yes	Anzai (Anzai Medical) (figure 1(d)) Bellows
Thermistor (2.1.3)	External	1D	No	Airflow temperature	Yes	Thermistor (non commercial)
kV/MV (2.2.2)	Internal	3D triangulated	Yes	Markers (prostate)	No	MSKCC (non commercial)
kV/kV (2.2.2)	Internal	3D triangulated	Yes	Markers (multi-site), vertebrae, Cranium	Dedicated machine	CyberKnife^®^ (Accuray) (figure 2(c) top)
				Markers (multi-site)	Dedicated machine	Vero (figure 2(c) bottom) (Brainlab and Mitsubishi, discontinued)
				Markers (multi-site)	Yes	RTRT (non commercial)
				Lung and liver tumours	Yes	Stereoscopic markerless monitoring (non commercial)
MV (2.2.3)	Internal	2D beam’s eye view	No	Markers, lung tumour	No	No online solution
		3D inferred		Markers (prostate)		
kV (2.2.3)	Internal	3D inferred	Yes	Markers (multi-site), vertebrae, bronchi, lung tumours	No	KIM (non commercial, online only for prostate) and sequential stereoscopic (non commercial, online only for vertebrae)
		6D inferred		Markers		KIM, not performed online
Hybrid (2.3)	Internal with correlation model	3D	Yes	Markers (multi-site), lung tumours	Dedicated machine	CyberKnife^®^ Synchrony (Accuray) (figure 2(c) top)
				Markers (multi-site)	Dedicated machine	Vero (figure 2(c) bottom) (Brainlab and Mitsubishi, discontinued)
				Markers (multi-site), cranium	Yes	ExacTrac (Brainlab) (figure 2(b))
				Markers (lung)	Yes	RTRT + Anzai (non commercial)
				Markers (liver)	No	COSMIK (non commercial)
Electromagnetic (2.4.1)	Internal	3D	No	Markers (multi-site)	Yes	Calypso (Varian) and rayPilot (MicroPos Medical, only prostate) (figure 5)
Ultrasound (2.4.2)	Internal	3D	No	Prostate, prostate bed	Yes	Clarity autoscan (Elekta) (figure 5(d))
				Soft tissues		Modified 4D ultrasound system (non commercial)
MR (2.5)	Internal	2D cine (any orientation)	No	Tissues	Dedicated machine	Unity (Elekta), MRIdian (ViewRay) (figure 2(d))

### 2.1. Surface imaging and respiratory monitoring

Respiratory monitoring can provide a surrogate for target motion in the thorax or abdomen and was proposed early on for gating (Kubo and Hill 1996). Audio-visual feedback to the patient may help improve breathing reproducibility. Surface imaging can provide direct target monitoring in the case of chest wall or breast irradiation. It is also considered to be a very reliable surrogate for intracranial targets. These methods are characterized by the ease of use and high temporal frequency without imposing additional imaging dose to the patient. However, for respiratory monitoring, they rely on the stability of the relationship between a certain respiratory level and the target position.

**Figure 1.**
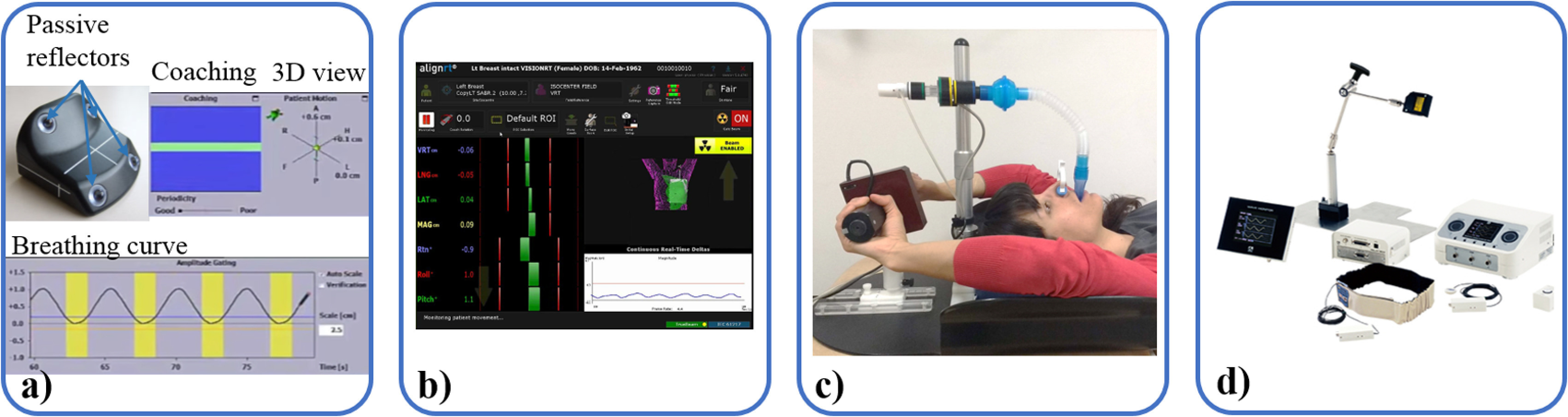
(a) Varian respiratory gating system uses an IR reflective marker. (Image provided courtesy of Varian) (b) align RT/OSMS is an optical surface monitoring device (image courtesy of Vision RT) (Vision RT, London, UK). (c) Elekta Active Breathing Coordinator (ABC) uses a spirometer to monitor lung volume (Courtesy, Helen McNair). (d) The Anzai pressure belt (Anzai Medical, Tokyo, Japan) monitors changes of the abdominal circumference.

**Figure 2.**
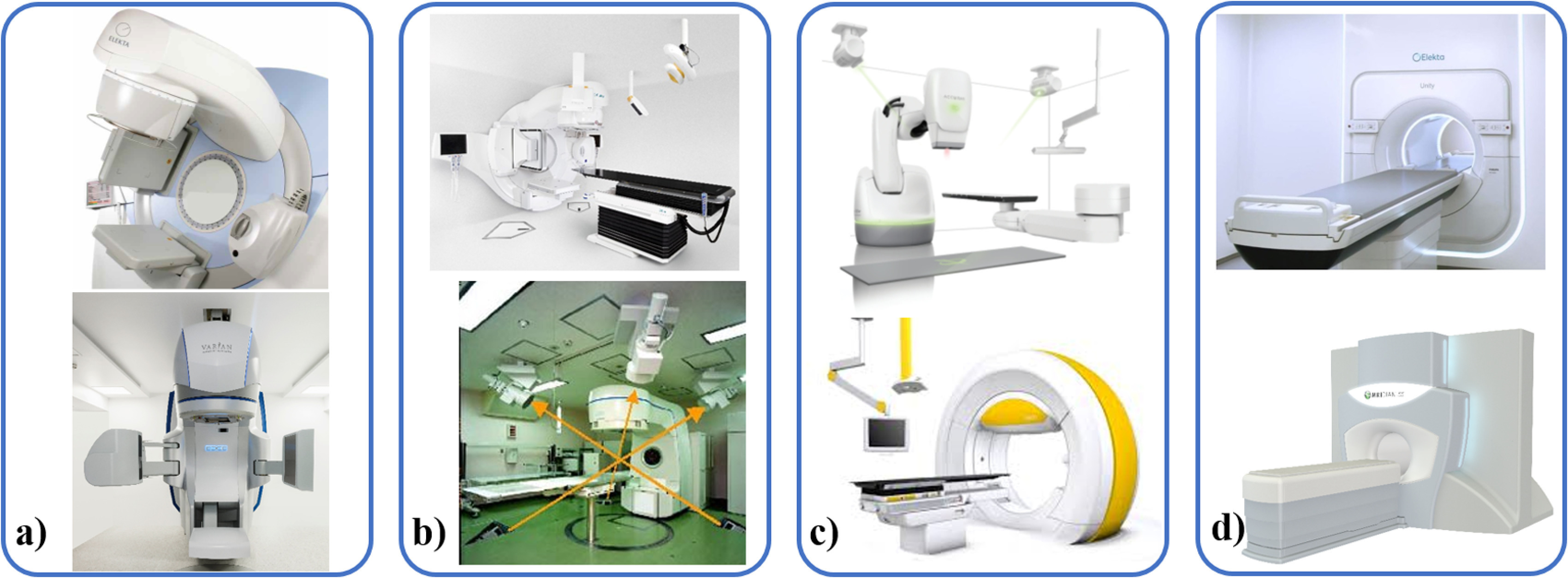
Systems for internal motion monitoring during RT delivery are shown. (a) Elekta (Elekta AB, Stockholm Sweden) (top, image courtesy of Elekta) and Varian (Varian Medical Systems, Palo Alto, CA) (bottom, image provided courtesy of Varian) standard linacs with a deployed MV imager opposite the treatment head and a perpendicularly mounted kV imaging system. (b) BrainLab ExacTrac (BrainLab AG, Feldkirchen, Germany) with stereoscopic kV imaging and external breathing monitoring (top, here mounted on an Elekta linac) and the RTRT system with four kV imaging systems (bottom, reproduced from https://rad.med.hokudai.ac.jp/en/research/treatment/tracking/ with permission). (c) The robotic CyberKnife^®^ (Accuray Inc, Sunnyvale, CA) system and Vero Gimbal (BrainLab and Mitsubishi Heavy Industries, Japan) incorporate stereoscopic kV imaging and external breathing monitoring. (d) Unity (top, image courtesy of Elekta) and MRIdian (Viewray Inc, Cleveland, OH) (bottom) are the two commercially available MR-guided linacs (see section 2.5).

#### 2.1.1. Infrared-based monitoring

Intracranial stereotactic radiosurgery (SRS) requires highly accurate treatment delivery. Infrared (IR)-based monitoring is a non-invasive alternative to fixed-pin systems where a coordinate frame is mechanically fixed to the patient’s skull (Lightstone *et al* 2005). This has led to the commercialisation of a number of 6 degree of freedom (DoF) systems using passive IR reflectors either mounted on the couch, a bite block, a thermoplastic mask, or the body of the patient (Bova *et al* 1997, Lightstone *et al* 2005, Willoughby *et al* 2012). Stereoscopic in-room cameras are used to monitor the IR reflector position, acting as surrogate for the tumour position (Jin *et al* 2008, Willoughby *et al* 2012). In addition, systems such as the ExacTrac 6D (Brainlab) and real-time position management (RPM) (figure 1(a)) can be used for respiratory gating of extracranial sites. These positioning systems are connected to a 6 DoF couch and are capable of beam interruption and patient repositioning during treatment with sub-millimetre accuracy (mean and SD of error) (Willoughby *et al* 2012).

RPM geometric accuracy was verified against fiducial marker (FM) trajectories for lung, liver and pancreas patients (Li *et al* 2012) and for lung patients treated in deep-inspiration breath-hold (DIBH) with visual feedback (Scherman Rydhög *et al* 2017). For RPM-guided left-sided breast DIBH treatments using multiple reflectors, Fassi *et al* (2018) reported a median residual 3D set-up error of 5.8 mm compared with kilovoltage (kV) images of implanted clips.

To reduce the internal–external correlation uncertainty, IR-based monitoring is often used in conjunction with x-ray monitoring as described in section 2.3. In addition, on True Beam linacs (Varian), the respiratory gating system can be used in tandem with the kV on-board imaging system (OBI) where kV imaging is used to verify the internal target anatomy at the beginning of the gated treatment window determined by the RPM signal. If the internal anatomy has changed, the treatment can be interrupted and the patient repositioned based on newly acquired volumetric imaging (Vinogradskiy *et al* 2018).

#### 2.1.2. Surface monitoring

Optical surface monitoring uses one or multiple high definition (HD) cameras to map the patient’s surface. AlignRT (Vision RT) (figure 1(b)) uses three such room-mounted cameras while Catalyst (C-RAD, Uppsala, Sweden) uses two room-mounted cameras. These systems project structured light patterns on the patient such that 6 DoF motion can be estimated (Willoughby *et al* 2012). Visible light from in-room lighting, the reflectivity and colour of patients’ clothing or skin tone can potentially affect the accuracy of surface mapping (Willoughby *et al* 2012). During treatment, the real-time detected patient surface can be compared with a reference surface, often obtained from the simulation CT. Typically one or more subsets of the surface can be selected as a region of interest (ROI) and are used to report the translation and rotation of the patient in real-time via registration to the reference surface. These systems can also replace skin tattoos for set-up and allow the use of less invasive fixation devices for SRS (Li *et al* 2011a, Pan *et al* 2012, Hoisak and Pawlicki 2018). Some integrated systems such as Align RT are able to automatically trigger beam-hold when the current surface does not match the reference surface. Re-positioning of the patient can be done in-room with immediate feedback from the system to guide the optimal match without the need for x-ray imaging.

Extracranially, surface guidance for intrafraction monitoring was mainly used for breast DIBH treatments (Tang *et al* 2014, Ma *et al* 2018). The main advantage of DIBH is the increased distance between the target volume and the heart resulting in lower dose to the heart and therefore lower rates of early toxicity (Zagar *et al* 2017). Using 3D surface mapping, Betgen *et al* (2013) evaluated the reproducibility of voluntary DIBH and found a systematic interfractional translation up to 5 mm.

#### 2.1.3. Other breathing surrogates

The airflow in and out of the lungs can be monitored using a spirometer which, in turn, is used to estimate the air volume inside the lungs at a given time point. The patient breathes through a mouthpiece, less leakage-prone than a mask (Wong *et al* 1999) and wears a nose clip to ensure that all the breathing occurs through the mouth (Hoisak *et al* 2004). In addition to the monitoring, a scissor valve can be added and used to maintain the air volume at a chosen level, therefore enforcing a breath-hold. This is known as active breathing control (ABC) and was first described by Wong *et al* (1999). A version by Elekta, under the name active breathing coordinator (ABC) (figure 1(c)) uses a balloon valve which prevents air-flow when inflated. ABC has been used for liver (Eccles *et al* 2006), left breast (Remouchamps *et al* 2003) and lung (McNair *et al* 2009) cancer patients. The main limitations for the use of ABC is the need for patient compliance, coaching sessions and good communication between the radiographer and the patient.

A thermistor measuring the air temperature may also be used to determine if the patient is inhaling or exhaling (Kubo and Hill 1996).

Pressure systems detect respiratory motion via the varying pressure in a belt around the abdominal section of the patient. The Anzai belt (Anzai Medical, figure 1(d)) is part of a respiratory gating system (Siemens) where a pressure sensor (30 mm diameter, 9.5 mm thickness) is inserted in the belt and outputs a binary 5 V signal to the linac depending on the gating window parameters.

### 2.2. kV and MV x-ray imaging-based methods

Image-based methods using kV and/or megavoltage (MV) x-ray imaging were a natural development from the concept of IGRT extending the use of in-room imaging from pre-treatment to intratreatment. As such, these methods represent a considerable body of work.

X-ray image-based methods come in different hardware configurations of stereoscopic or monoscopic imaging (figures 2(a)–(c)) and can be combined with external monitoring (section 2.3). Common to all image-based methods is the need for image processing to retrieve the target position information from the planar image or set of images. The latency of x-ray image-based methods includes the image acquisition time and the processing time (Fledelius *et al* 2011).

#### 2.2.1. Marker implantation and real-time segmentation in kV and MV images

**Figure 3.**
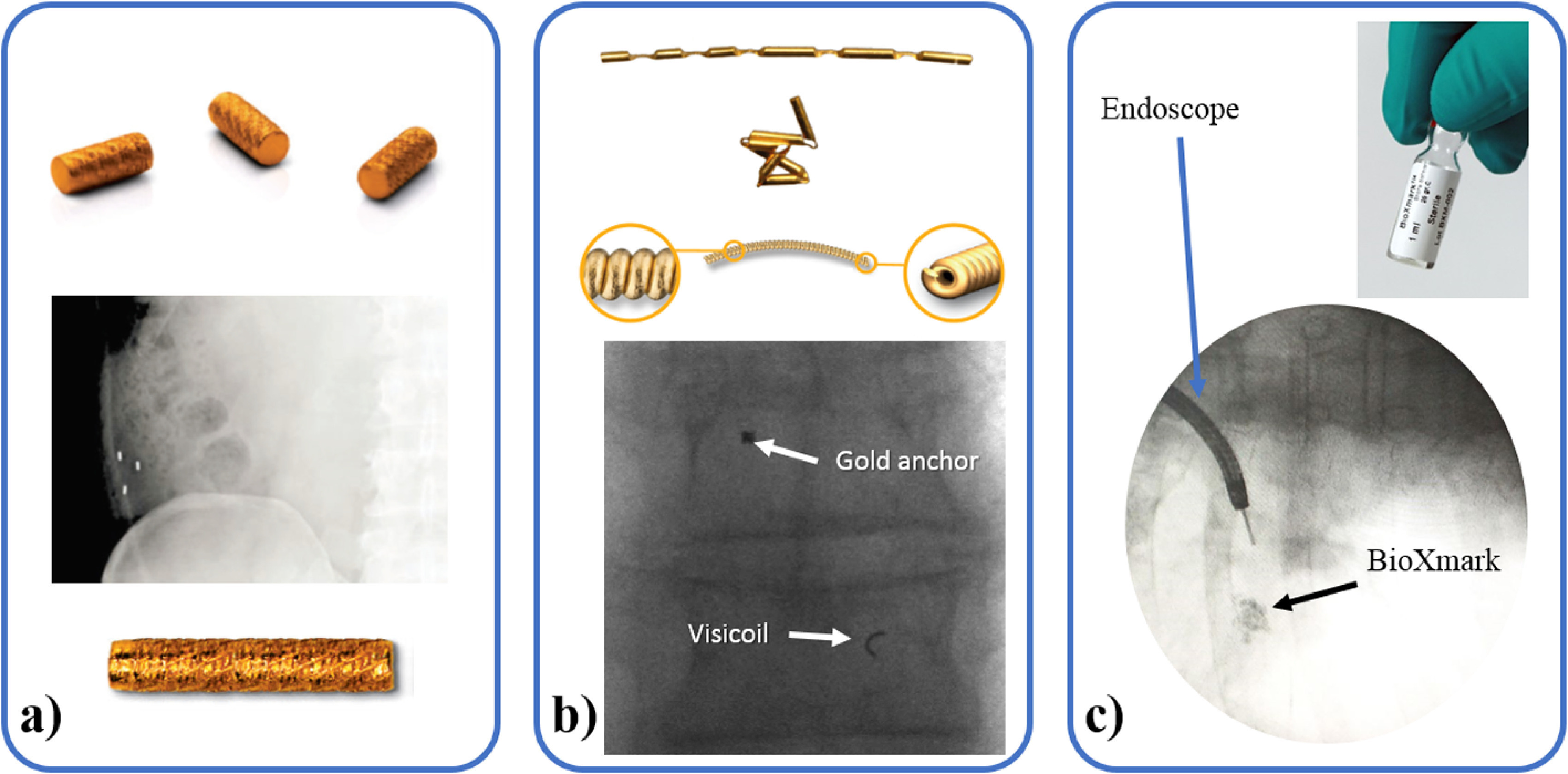
Examples of FM. (a) 3 mm-long gold markers (civco, diameter between 0.8 and 1.2 mm) (top) can be implanted in any soft tissue (middle) for image guidance. The similar 5  ×  1 mm CyberMark^™^ was developed specifically for use with CyberKnife^®^ (bottom) (civco Radiotherapy, Coralville, IA). (b) Gold anchor (Naslund Medical, Sweden) (diameter of 0.28 or 0.4 mm) (top) and Visicoils (IBA dosimetry, Barlett, TN) (diameter between 0.35 and 1.1 mm) (middle) take an arbitrary shape once implanted (bottom). (c) The liquid fiducial BioXmark (Nanovi, A/S, Denmark) before (top) and after endoscopic assisted implantation (bottom).

Most commonly, high contrast implanted FMs (figure 3) act as surrogate for the tumour position due to poor soft tissue contrast. FMs (figure 3(a)) are routinely implanted in the prostate for pre-treatment image guidance but may also be implanted percutaneously in the liver, pancreas and lungs or bronchoscopically in the peripheral lung (Shirato *et al* 2007) and in mediastinal lymph nodes (Schmidt *et al* 2016). Endoscopic implantation is possible into or near the digestive tract (Fukada *et al* 2013) while spinal and paraspinal lesion implantations are performed surgically (Shirato *et al* 2007). Endovascular coils have also been used as markers for lung tumours (Prévost *et al* 2008). Thinner markers that can take an irregular shape (figure 3(b)) may be preferred to regularly shaped markers to limit artefacts in reconstructed volumetric images or the risk of migration or implantation complication (Hanazawa *et al* 2017, Castellanos *et al* 2018). Liquid FMs such as Lipiodol (Guerbet, France) (Rose *et al* 2014) or BioXmark (figure 3(c)) allow for a personalized injected volume, reduced artefacts in reconstructed volume images and reduced dose perturbation for particle therapy at the cost of lower contrast in x-ray projection images.

For any treatment guidance or adaptation based on intrafraction monitoring, markers must be segmented automatically in real-time, which is more difficult in MV images that have inherently lower contrast than kV images (Mao *et al* 2008, Lin *et al* 2013) and may have markers close to or outside the field edge (Fledelius *et al* 2014, Poulsen *et al* 2014, Hunt *et al* 2016). MV scatter onto the kV imager may also degrade the kV image quality (Luo *et al* 2008, Fledelius *et al* 2014) but can be efficiently reduced using triggered read-out to eliminate the accumulated MV scatter before each kV image acquisition (Poulsen *et al* 2015a).

Cylindrical or spherical markers can be segmented in real-time in kV or MV projections using simple parametric templates (Tang *et al* 2007, Mao *et al* 2008, Marchant *et al* 2012, Fledelius *et al* 2014). Arbitrarily shaped markers or marker groups require more complex templates that can be generated semi-automatically using breath-hold computed tomography (CT) scans (Regmi *et al* 2014) or fully automatically using pre-treatment cone-beam CT (CBCT) projections (Bertholet *et al* 2017, Campbell *et al* 2017b). The segmented marker position is typically selected as the one with the highest normalized cross-correlation coefficient between the 2D template and a pre-defined ROI of the projection. There will always be a maximum in the normalized cross-correlation hence causing segmentation error if the marker is outside of the ROI. A larger ROI increases the chances that the marker is inside the ROI, but the computation time increases linearly with the ROI area and the template area, and a larger ROI increases the risk of mistaking the marker for some other structure in the image (Fledelius *et al* 2014). Suitable ROIs result in a typical processing time below 10 ms per marker per image (Mao *et al* 2008, Fledelius *et al* 2014). Low cross-correlation coefficients also allow to detect potentially erroneous segmentation in template-based methods (Tang *et al* 2007, Fledelius *et al* 2014, Bertholet *et al* 2017) (table 2).

**Table 2.** Properties of the marker segmentation algorithms discussed in section 2.2.1.

Method	Marker shape	Site (patient number)	Image type	Template generation	Manual input needed	Automatic error detection
Fledelius *et al* (2014)	Cylindrical	Liver (13)	CBCT, kV, MV	Automatic	No	Yes—rejected segmentation
Mao *et al* (2008)	Spherical, Cylindrical	Prostate (5)	kV, MV	Automatic	No	No^a^
Tang *et al* (2007)	Cylindrical	Liver (2)	kV	Automatic (from library)	Yes (initialization)	Yes—terminates segmentation
Marchant *et al* (2012)	Cylindrical	Pancreas (2), prostate (1)	CBCT	Gaussian kernels	Yes (initialization)	No^a^
Regmi *et al* (2014)	Arbitrary (Visicoil), Cylindrical	Pancreas (4), Gastrointestinal junction (6), lungs (1)	CBCT	From breath-hold CT	Yes (template generation pre-treatment)	No
Bertholet *et al* (2017)	Arbitrary (Visicoil), Cylindrical	Thorax (12), Abdomen (28)	CBCT	Automatic	No	Yes—rejected segmentation
Campbell *et al* (2017b)^b^	Cylindrical marker group	Pancreas (15)	CBCT	Automatic	No	No^a^
Lin *et al* (2013)	Cylindrical	Prostate (2)	MV	No	Yes (manual selection of training sample at fraction 1)	No
Wan *et al* (2016)^b^	Arbitrary (visicoil, embolization coil), cylindrical (gold, Calypso)	Abdomen (34), Lung (5)	CBCT	No	No	No

a Methods designed to have a 100% detection rate.

b Not fully demonstrated in real-time.

Template-free methods were also proposed using machine-learning with manually labelled data from the first treatment fraction as training dataset (Lin *et al* 2013) or using a dynamic programming (DP)-based method (Wan *et al* 2014, 2016). Due to the post-processing nature of the DP-based algorithm, it has not been used in real-time to date. However, owing to the fast processing time, a pre-treatment imaging data set could be acquired to initiate detection and intra-treatment images could be appended to the data set as they are acquired for real-time segmentation.

Table 2 summarizes the properties of selected methods. Note that accuracy results are not presented here. A fair comparison of segmentation algorithms is particularly difficult given the variety of image quality, marker type, treatment site, and ground truth data used for the evaluation.

FMs and their implantation represent an added cost and toxicity risk. Percutaneous implantation was linked to a risk similar to conventional percutaneous biopsy in lung, pancreas and liver (Kothary *et al* 2009) with pneumothorax as the most common complication. For trans-rectal implantation in the prostate, the main risk is urinary tract infection. However, it may be minimized by the use of thin markers requiring a small needle (Castellanos *et al* 2018). The use of markers also implies delays in the treatment due to the implantation itself but also often a waiting time between implantation and planning CT to let markers stabilize although a delay between implantation and planning CT was found to be unnecessary in liver patients (Worm *et al* 2016). Other limitations include marker migration and changes in the tumour position relative to the markers due to tissue deformations. Especially in the liver where markers are often implanted outside of the tumour to avoid tumour seeding during percutaneous implantation, an increased target-surrogate distance has been linked to a reduced targeting accuracy (Seppenwoolde *et al* 2011). For transbronchial implantation in the lungs, Ueki *et al* (2014) reported a residual intrafractional variation of the tumour position with respect to the markers of 1.5 mm in the SI direction. Shirato *et al* (2007) reported on the Hokkaido group experience in marker implantation in multiple sites with multiple techniques and reported successful implantation in 90 of 100 lesions without any serious complication. They observed that there is a learning curve among endoscopists regarding fixation rate for implantation in the bronchial tree and that the relationship between the markers and tumour can change significantly after two weeks. To avoid the risk, cost, and uncertainty related to the use of FMs, markerless monitoring in kV and MV images may be used for certain sites (sections 2.2 and 2.3).

#### 2.2.2. Stereoscopic imaging methods

Real-time x-ray imaging is limited to 2D localization information. Ideally, stereoscopic kV imaging is used to determine the target position via triangulation with high accuracy. However, this requires additional equipment.

##### 2.2.2.1. The CyberKnife^®^ system

The CyberKnife^®^ system (figure 2(c) top) was developed for frameless cranial SRS radiosurgery in the 1990s (Adler *et al* 1997) and shortly thereafter modified to treat extracranial sites (Murphy *et al* 2000). The system consists of two ceiling-mounted kV sources, two opposed floor-mounted flat panel detectors (FPD) and automatic image processing software controlling a robotic 6 MV-linac in real-time. The robotic linac can re-align the treatment beam with 6 DoF in a non-isocentric manner, therefore being the first dedicated treatment machine combining motion monitoring and tracking. The system can monitor the target position with 6 DoF by co-registering two simultaneously acquired intra-treatment radiographs to CT-generated digitally reconstructed radiographs (DRR). The first clinical applications were for markerless monitoring for cranial SRS (Adler *et al* 1997) and for cervical spine treatment in one patient (Murphy *et al* 2000). Cranium and spine are well suited for markerless monitoring where the high contrast of the bony anatomy allows for reliable registration. Intratreatment radiographs can only be acquired every 10 or 20 s, which is insufficient to resolve breathing motion. For respiratory motion, the x-ray monitoring is combined with continuous optical monitoring as described in section 2.3. Although insufficient to resolve respiratory motion, stereoscopic imaging on the CyberKnife^®^ system has been extensively used to monitor prostate motion during SBRT (Friedland *et al* 2009, King *et al* 2012).

##### 2.2.2.2. The RTRT system

High frequency intra-treatment stereoscopic imaging for monitoring was pioneered in the late 1990s by Shirato *et al* (1999) who installed an orthogonal x-ray imaging system in the treatment room of a conventional linac creating the real-time tracking radiotherapy (RTRT) system (Shirato *et al* 2000). Note that the RTRT system does not perform tracking in the sense of following the tumour with the treatment beam. Instead the position of a FM is monitored in real-time and the treatment beam is gated (Shirato *et al* 1999). The imaging part consists of four x-ray sources in the floor corners (superior right and left and inferior right and left), with corresponding ceiling-mounted detectors. The linac and the imaging system isocenters coincide and only two orthogonal x-ray systems with unobstructed views are used at a time. The linac and the kV imaging system pulses are synchronized such that the kV images are free from MV scatter. Thirty kV image pairs are acquired per second and used to detect a spherical or Visicoil (Hanazawa *et al* 2017) FM using a simple template matching algorithm. Beam interlocks are set if the cross-correlation coefficient is too low or if the line of sight of the marker in the two imagers are further apart than 1.5 mm. The high monitoring rate of the RTRT system has permitted to extensively study tumour motion in various anatomical sites (Seppenwoolde *et al* 2002, Kitamura *et al* 2003, 2002, Ahn *et al* 2004, Hashimoto *et al* 2005, Shirato *et al* 2007, Kinoshita *et al* 2008).

Shiinoki *et al* (2017) proposed to incorporate an RTRT-like system on a Varian linac: the SyncTraX system where only two cameras are used but can be set at three possible positions to ensure un-obstructed view. Berbeco *et al* (2004) also proposed a prototype integrated radiotherapy imaging system (IRIS). Although IRIS was not used clinically, the idea of a gantry-mounted stereoscopic imaging system was later commercialized as the Vero system.

##### 2.2.2.3. The Vero system

The Vero system (figure 2(c) bottom) was described by Kamino *et al* (2006) and consists of an O-ring gantry with a small gimbals-supported linac head. Two kV sources and opposite FPDs are mounted in the O-ring gantry at 45° with respect to the treatment beam and an EPID panel allows beam’s eye view (BEV) imaging. Pan and tilt of the gimbals as well as skew angle of the gantry allow the treatment beam to track targets affected by respiratory and cardiac motion. The Vero system is used to treat patients with real-time tumour tracking (RTTT) based on a hybrid monitoring method (see section 2.3). However, Dhont *et al* (2017) used the 20 s stereoscopic imaging session (at 11 Hz) used for an external correlation model (ECM) building to investigate short and long-term variations in breathing induced motion for 19 lung and 18 liver lesions bearing one Visicoil marker each. Substantial intrafractional drift (SI) was observed for both treatment sites with mean  ±  SD values of 4.1  ±  1.7 mm and 3.0  ±  1.2 mm for lung and liver lesions respectively. Note that the Vero system is no longer commercially available.

##### 2.2.2.4. Markerless stereoscopic monitoring

In addition to the XSight Lung application described in section 2.3, the other markerless monitoring application that has been clinically used is the work of Mori *et al* (2016). They have used this approach to treat both lung and liver cancer patients, making this the first application of markerless monitoring for liver cancer. They use a stereoscopic imaging system to acquire a series of patient images throughout the respiratory cycle. Their markerless tumour monitoring method uses multi-template matching and machine-learning algorithms, template images and a machine-learning dictionary file. Learning is performed for each patient based on the pre-treatment images. Once a model has been built and verified, the model is applied to process the images in real-time to determine the tumour position. The markerless monitoring system derives the beam pause function of their carbon ion treatment beam, enabling gated treatment.

##### 2.2.2.5. Combined kV/MV

On a conventional linac, MV imaging may complement kV imaging for triangulation of the target position. However, due to the low contrast of MV imaging, pre-processing techniques are often required. Hunt *et al* (2016) proposed to combine MV digital tomosynthesis (DTS) with kV imaging during volumetric arc therapy (VMAT) for patients with prostate cancer using conventional linacs (figure 2(a)). The method was evaluated in phantom experiments and for three prostate patients treated with VMAT, each having three implanted cylindrical fiducials. MV images were acquired continuously at ~9.5 Hz and arcs between 2 and 7° were used for MV-DTS while kV images were acquired every 20°. MV-DTS reduces the visibility of out-of-plane objects such as bony anatomy, however, a greater arc may result in blurring of the fiducials due to prostate motion and therefore hinder marker visibility. Single MV images or MV-DTS were paired with the corresponding kV image, FMs were segmented and their 3D positions were determined by triangulation. In patients, motion monitoring results were validated against manual FM selection in single MV images triangulated with the closest kV image (ground truth position). Marker detection failures increased with the span of the MV-DTS due to MLC leaves obstructions of the markers in the MV images. The total processing time for fiducial detection in a 4° MV-DTS was 1.1 s of which 0.6 s was the MV-DTS reconstruction time.

The authors addressed the marker detection failure in MV images by developing an automatic plan optimization strategy ensuring that at least one fiducial was always visible (Zhang *et al* 2016). Exposing one fiducial was feasible without loss of plan quality. The method has now been clinically implemented to treat more than 110 prostate patients with gating (Keall *et al* 2018b). The same group recently extended the method to markerless kV/MV lung tumour monitoring by registering kV and MV images to CBCT projections acquired at the same gantry angle (Zhang *et al* 2018).

#### 2.2.3. Monoscopic imaging methods

##### 2.2.3.1. KV monoscopic imaging

On a standard linac, the kV imaging system is mounted perpendicularly to the linac head (figure 2(a)). Algorithms are thus used to infer motion in the unresolved dimension. kV images have better contrast than MV images, allowing more reliable detection of the target (FM or tumour) position in real time. Furthermore, the kV field-of-view can be selected to cover the target independently of the treatment beam shape, and kV images may be acquired prior to treatment onset as a training dataset for model building and motion prediction.

When a point target is projected onto an x-ray imager it is known to be located somewhere on the ray line between the projection point and the x-ray source. Real-time monoscopic target localization in general uses the projected target position in a sequence of training images from different angles to establish a model that allows estimation of the unresolved target position along the ray line (and thus the 3D position) in a new image. The model is assumed to be constant over a certain time such that it can be established by partial information from training images acquired at different times.

A very simple model is to neglect the motion taking place in the unresolved direction. The unresolved target position in the current image can then be determined by triangulation as the position on the ray line of a training image that is closest to the ray line of the current image. The triangulation can include several training images, possibly with different weights and can be rejected if the ray line is more than a certain threshold distance from the ray line of the current image or other training images. This is the idea behind Sequential Stereo (Varian Medical System), which was recently used for online real-time 3D spine localization during VMAT SBRT delivery (Hazelaar *et al* 2018c). Sequential Stereo (Van Sörnsen De Koste *et al* 2015) and similar methods (Regmi *et al* 2014) can be used in the presence of respiratory motion provided that training images at the same breathing phase and with ray lines sufficiently close to the current image are available for the triangulation. This requirement can be avoided, e.g. by assuming a confined 3D target trajectory defined by the mean 3D position in two (Park *et al* 2012) or more (Becker *et al* 2010) respiratory phases as estimated by back-projecting sets of phase-sorted training images. The unresolved position of the current image is then estimated as the position closest to the confined 3D target trajectory.

Another approach is to establish a 3D probability density function (PDF) for the target position from a sequence of training images and estimate the unresolved position of the current image as the expectation or maximum value of the 1D PDF along the ray line. One possibility is a 3D Gaussian PDF determined from the projected target positions by maximum likelihood estimation (Poulsen *et al* 2008a). Another possibility is a Bayesian approach, where the 3D PDF is a product of individual contributions from training images that have uniform probability distributions along the ray line and exponential decay away from the ray line (Li *et al* 2011b). The PDF-based methods can be used for both respiratory motion and non-periodic motion such as prostate motion.

A drawback of PDF-based methods is that the 3D-PDF must be rebuilt periodically to capture the possible changes in the distribution of motion (correlation or covariance of the 3D motion). The Kalman filter approach can overcome this drawback by iteratively re-estimating the posteriori function without solving all the parameters of the PDF (Kalman 1960, Shieh *et al* 2017). The Kalman filter framework implicitly assumes a Gaussian distributions which is computationally more efficient than other probabilistic approaches.

For respiratory motion, another approach is to exploit interdimensional motion correlation to model the unresolved LR and AP target positions as a function of the resolved SI position (Chung *et al* 2016). The parameters of the correlation model are fitted to the training images in an iterative way to account for the position dependent scaling factor between room coordinates and imager coordinates. When the correlation model is established, the full 3D position of the current image is estimated from the observed SI position. When an external respiratory signal is available a related approach is to establish an ECM of the target position along all three axes as function of the respiratory signal (Cho *et al* 2010) (see section 2.3).

A direct comparison between the different monoscopic methods is difficult since the performance depends on several factors such as the image sequence, motion trajectory, and possible model parameters. However, a recent comparison reported that the Gaussian and Bayesian PDF, the Kalman filter and the interdimensional motion correlation methods all had sub-millimetre accuracy (mean and SD of error) with the Gaussian PDF methods being the most precise (Montanaro *et al* 2018). One important limitation of this work is that segmentation, hence, 2D target information, was assumed to be perfect. In the presence of noise and segmentation errors, lower accuracy is expected.

The most widely used method is Kilovoltage Intrafraction Monitoring (KIM) which integrates the Gaussian PDF method for 3D motion estimation with template-based marker segmentation and has been used both retrospectively (Ng *et al* 2012) and prospectively (Keall *et al* 2015) for prostate cancer patients. In addition, similar systems were used to retrospectively estimate intrafraction motion of liver tumours for VMAT treatments (Poulsen *et al* 2014) and pancreas tumours in daily CBCT (Jones *et al* 2015). For these clinical applications, the tumour location is implicitly inferred by calculating the positions of the implanted gold FM. KIM accuracy has so far been evaluated against post-treatment triangulation, reporting sub-millimetre accuracy (mean and SD of error) in both retrospective analysis (Ng *et al* 2012) and prospective motion monitoring with beam gating and couch-shifts (Keall *et al* 2016). Recently, the KIM system has been extended for six degrees of freedom (DoF) motion monitoring in prostate patients (Nguyen *et al* 2017b). Measurements with a phantom show that sub-millimetre and sub-degree accuracy can be achieved for both prostate and lung motion traces (Kim *et al* 2017). In future applications, this can be replaced by direct 6 DoF motion estimation from 2D projection data to avoid the intermediary 3D estimation step (Nguyen *et al* 2017a). To date, more than 120 prostate patients have been treated with KIM monitoring.

Markers implanted into or adjacent to the tumour give the treatment team high confidence in the treatment targeting. However, as discussed in section 2.2.1, markerless approaches are highly desirable to avoid the added cost, risk and geometric uncertainty related to the use of FMs. Given the high-density contrast in the lungs where the lung tissue density is approximately 20% of the tumour and surrounding tissue density, lung cancers are an ideal area to explore with x-ray image guidance.

Early work by Berbeco *et al* (2005a) used fixed angle kV beams for tumour position analysis to determine when to gate the radiation beam. More recently a number of groups have developed sophisticated methods to determine the lung tumour position from these images (Lewis *et al* 2010, Ren *et al* 2014, Zhang *et al* 2014a, Shieh *et al* 2017, Hazelaar *et al* 2018a). Though most of the work to date has been with single energy images, the ability to acquire dual energy x-rays can help with bone signal subtraction for enhanced soft tissue contrast (Patel *et al* 2015). Of note a recent study demonstrated bronchus monitoring on phantom and retrospective patient images (Hazelaar *et al* 2018d). Monitoring of the bronchus is interesting as it is an avoidance structure as well as a surrogate for the target position therefore allowing simultaneous tumour and normal tissue monitoring.

##### 2.2.3.2. MV monoscopic imaging

MV imaging using the treatment beam itself as a source and an electronic portal imaging device (EPID) is known as BEV imaging and does not add imaging dose to the patient. In addition, although MV BEV monitoring is not 3D, it does yield motion measurements in the two dimensions most sensitive to motion for photon radiotherapy, i.e. perpendicular to the treatment beam. However, MV imaging has poorer soft tissue and marker contrast than kV imaging, can only be used when the treatment beam is on, and the field of view is limited to the treatment beam and affected by the amount of beam modulation. BEV MV imaging was proposed both for marker and markerless monitoring.

In pioneering work, Deutschmann *et al* (2012) used MV imaging of four markers implanted into the prostate to estimate the positional and rotational pose of the prostate and adapt the treatment accordingly. The prostate position was determined prior to each IMRT segment, and the segment positions for the IMRT treatment were adjusted accordingly without needing to adjust the couch position. To achieve this, a record-and-verify system with integrated treatment planning system had to be developed. This method was successfully applied in over 1000 fractions for 39 prostate cancer patients. The authors found over 2 mm prostate drifts in 82% (833) of the fractions. Target rotation of  >12° was found for 10% of fractions. They concluded that the inter- and intrafraction motion measurements and adaptation enabled safe margin reduction. Though 2D motion measurements in BEV may be sufficient for photon radiotherapy applications, Azcona *et al* (2013) applied a 2D to 3D trajectory reconstruction algorithm (Li *et al* 2011b) to the motion measured in clinical MV prostate images to establish the 3D target position during treatment.

MV BEV motion monitoring was experimentally implemented and demonstrated with MLC tracking for SBRT delivery in pigs with an implanted stent in the lung (Poulsen *et al* 2012a). In addition, it was used retrospectively for markerless monitoring on clinically acquired lung images (Richter *et al* 2010, Aristophanous *et al* 2011, Rottmann *et al* 2013).

#### 2.2.4. Imaging dose

As reported in the AAPM TG75 report, a substantial limitation of kV imaging-based motion monitoring is the added imaging dose to the patient, especially at the skin surface (Murphy *et al* 2007). A kV image from a standard linac delivers 1–3 mGy per image depending on the technique. A total added imaging dose of 2–10 mSv was estimated for KIM-guided prostate RT at 1 Hz and, for comparison, the dose typically delivered by one pelvis CBCT scan was 4.3 mSv (Ng *et al* 2013). On the RTRT system (Shirato *et al* 2004) the skin dose from one fluoroscope was estimated to 29–1182 mGy h^−1^ and was highly dependent on kV peak and pulse duration but less so on skin-isocenter distance. Transient or main erythema can appear with an imaging dose of 2000 mGy or 6000 mGy respectively (Murphy *et al* 2007). Skin dose is therefore non-negligible for long IMRT treatments with the RTRT system. Depth dose at 5 cm was up to 58% of the peak dose and may also become a concern in IMRT treatments. Reduction of field size is an important but insufficient measure to reduce the dose, since the same area will receive the same skin dose every day. In a gantry mounted system, the source to detector distance is shorter than for the RTRT system which reduces exposure by a third compared to that of the RTRT system for a similar dose at the imager. The most direct way to reduce exposure remains reducing the imaging frequency as implemented in later generations of the RTRT system (Shirato *et al* 2004) or using hybrid monitoring.

### 2.3. Hybrid methods

**Figure 4.**
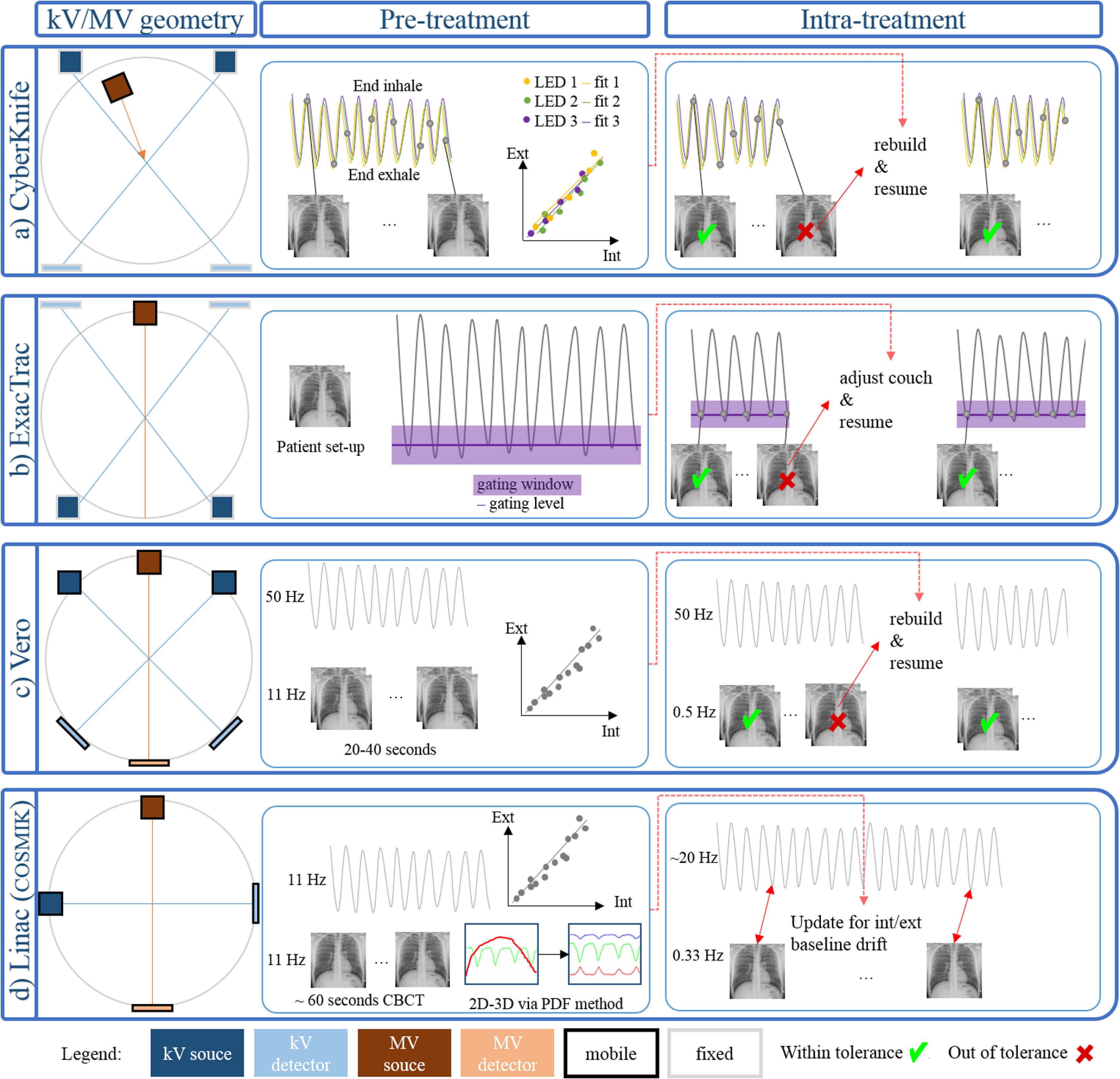
Schematic overview of the geometry, pre-treatment model building and intra-treatment monitoring for the hybrid monitoring platforms. Note that the ExacTrac kV imaging system is non-coplanar at 60° angle (figure 2(b)) and the MV source of the CyberKnife^®^ can move non-isocentrically.

Respiratory monitoring (section 2.1) is a poor surrogate for the position of internal targets (Hoisak *et al* 2004, Li *et al* 2012). To address this shortcoming, intrafraction imaging of FMs may be used to verify external monitoring (see section 2.1.1). In addition, hybrid monitoring methods were developed specifically to combine respiratory monitoring with sparse imaging for internal monitoring. The general workflow includes a pre-treatment training phase of simultaneous internal and external monitoring where an ECM is built that relates the internal motion to the external motion. During treatment, the internal position is estimated from the external signal. Sparse imaging is used to verify the stability of the ECM and/or trigger an ECM update or rebuild if needed (see section 2.3.6). Figure 4 illustrates the kV geometry and gives a schematic overview of the pre-treatment model building and intra-treatment monitoring on the various platforms. Note that in all cases, the external monitoring (not shown on figure 4) is provided by ceiling mounted cameras and reflective or emitting markers on the patient chest and/or abdomen (section 2.1).

#### 2.3.1. The CyberKnife^®^ Synchrony system

In addition to the robotic linac and kV imaging system of the CyberKnife^®^ system (see section 2.2.2), Synchrony comprises a vest fitted with light emitting diodes (LED) markers and three ceiling-mounted cameras to monitor external motion at 20–40 Hz (Ozhasoglu *et al* 2008). Prior to treatment, at least eight x-ray pairs are acquired at different breathing phases (including end-inhale and end-exhale) and used to triangulate the fiducial maker positions (figure 4(a)). The external motion is continuously recorded, and an ECM is built that relates the internal FM motion to the external marker motion (see section 2.3.6). During treatment, the ECM is used to infer the marker positions and re-align the treatment beam. In addition, new x-ray pairs can be acquired about every 30 s to directly determine the FM positions by triangulation. The model can be updated on the fly in case of small errors or completely rebuilt using a new set of eight x-ray pairs after treatment interruption.

Hoogeman *et al* (2009) analysed the log files for the treatment of 44 lung cancer patients on the CyberKnife^®^ Synchrony system and calculated the correlation error as the difference between the estimated target positions and the actual target position in the intra-treatment images. They found a sub-millimetre population mean error (mean of the SDs) in each direction and no difference in correlation model error between centrally or peripherally located tumours.

Bibault *et al* (2014) reported on markerless lung tumour monitoring using the Synchrony system for 51 patients. The method is known as Xsight Lung Tracking System and allows to use the DRR method (see section 2.2.2) for lung tumours larger than 15 mm located in the apex and peripheral lung region and further than 15 mm away from major vessels and ribs. Another detectability criterion was that the projection of the tumour onto the spine must be at an angle different from 45°.

#### 2.3.2. The ExacTrac system

The ExacTrac system (figures 2(b) and 4(b)) combines an IR camera system with two floor-mounted kV sources and opposite ceiling-mounted detectors (Willoughby *et al* 2006a). Between five and seven external IR reflective markers are placed on the patient and detected by ceiling-mounted cameras (see section 2.1.1). An IR reflective star is placed on the couch and used for automatic couch adjustments. During treatment, when the external signal matches the reference gating level, an x-ray image pair is acquired and the 3D triangulated position of a FM is compared with its reference position. If there is a discrepancy larger than a set tolerance, the beam is switched off and the couch position is adjusted. Willoughby *et al* (2006a) and Verellen *et al* (2006) reported on the initial clinical experience with 11 and three lung cancer patients respectively. A 6D fusion option was later implemented to allow 6 DoF localization from the kV imaging system (Jin *et al* 2008).

In cranial SRS, reflective IR markers placed on a thermoplastic mask may be used for intrafraction monitoring (see section 2.1.1). However, the masks are slightly elastic and patients may still move within the mask. On the ExacTrac system, x-ray pairs can be acquired and 6 DoF position correction is obtained by 2D/3D image registration with planning DRRs. Radiograph pairs can be acquired for verification pre- and post-treatment (Gevaert *et al* 2012).

#### 2.3.3. The Vero system

The Vero system described in section 2.2.2 includes an ExacTrac IR camera system. At the start of treatment, simultaneous stereoscopic kV imaging at 11 Hz and external IR monitoring at 50 Hz are performed in a 20–40 s training session to build an ECM (figure 4(c)). During treatment, the internal target position is determined from the ECM and stereoscopic images are acquired every 2 s. A ROI corresponding to a 3 mm tolerance radius around the predicted FM position is shown and the user can decide to terminate the session if the tolerance is systematically exceeded. Depuydt *et al* (2014) reported on the first ten liver and lung SBRT patients treated on the Vero RTTT system. The ECM building took an average of 2.7 min and was valid for an average (range) of 6.9 min (2.7–17.4 min). Significant cranial and posterior drift were observed for the IR and internal SI signal at the beginning of treatment suggesting that the drift was due to patient relaxation. Following a similar analysis for ten lung cancer patients, Akimoto *et al* (2013) recommended frequent model updates to avoid large baseline drift-related errors.

#### 2.3.4. RTRT with optical Anzai Belt

An RTRT system was installed at the Nippon Telegraph and Telephone corporation Hospital in Sapporo, Japan. However, this system only had two kV imagers which may have an obstructed view at certain gantry angles (Berbeco *et al* 2005b). The system was therefore supplemented by an external optical system (Anzai Medical) using a laser source and detector on an extendable arm placed on the treatment couch. Berbeco *et al* (2005b) investigated the residual motion for eight lung cancer patients treated with respiratory gating. Amplitude-based gating had slightly lower residual motion than phase-based gating for irregular breathing. Beam-to-beam and day-to-day variations were observed that warrant an adjustment of the gating window during the course of treatment, preferably based on online internal imaging.

#### 2.3.5. COSMIK

Bertholet *et al* (2018) implemented hybrid monitoring on a standard linac using Combined Optical and Sparse Monoscopic Imaging with Kilovoltage x-rays (COSMIK, figure 4(d)). The method was developed as a hybrid alternative to KIM and therefore uses a similar monoscopic imaging technique and the RPM (Varian) as external monitoring device. COSMIK uses a pre-treatment CBCT both for patient set-up and as a training data set for ECM building. The FMs are automatically segmented in the CBCT projections (Bertholet *et al* 2017) and their 3D trajectories are estimated using the Gaussian PDF method (Poulsen *et al* 2008a). The 3D FM trajectories are used for automatic patient set-up (Worm *et al* 2012) and to fit an augmented linear ECM (Ruan *et al* 2008). During treatment, the internal FM positions are estimated from the continuous external signal using the ECM. kV images are acquired every 3 s, the FMs are segmented and their 3D positions are estimated. The ECM is updated based on the last three images for baseline drift between the internal and external signal. COSMIK can be used for non-coplanar fields without imaging, using the latest updated ECM. COSMIK was validated in phantom experiments and simulations and used on 14 liver SBRT patients treated with implanted FMs without motion mitigation. COSMIK was more recently combined with real-time 4D dose reconstruction (Ravkilde *et al* 2018, Skouboe *et al* 2019).

#### 2.3.6. Correlation models and update strategies

Hybrid methods with ECM updates are more accurate than monitoring based on respiratory signals alone (Malinowski *et al* 2013, Poels *et al* 2014, Bertholet *et al* 2018) but less accurate than continuous kV imaging and they cannot be used to monitor non-correlated internal motion such as seen in the prostate. Similar accuracy is achievable on specialized equipment and standard linacs because the accuracy is limited by the use of an ECM rather than by the way (stereoscopic or monoscopic kV imaging) that the ECM is being established (Cho *et al* 2010, Bertholet *et al* 2018). Despite the lower accuracy related to the use of an ECM, hybrid monitoring presents certain advantages over continuous kV imaging such as reduced imaging dose, shorter latency, continuous monitoring even during beam-off time, robustness to missing or erroneous marker segmentation and compatibility with non-coplanar treatment fields.

External/internal correlation and ECMs are therefore central to the use of hybrid methods. Several studies have investigated the correlation between breathing and target motion, the stability of that correlation, ECMs of different forms, and update strategies (McClelland *et al* 2013). The external motion is often ambiguously related to the internal motion due to hysteresis where the same external position results in different internal positions during inhale and exhale. Linear or quadratic models cannot model hysteresis but may be combined with state augmentation using a time delayed sample (Ruan *et al* 2008) or the first temporal derivative of the external position (Depuydt *et al* 2013).

On the CyberKnife^®^ system, the hysteresis is addressed by using two quadratic functions without state augmentation: one for the inhale phase and one for the exhale phase (Seppenwoolde *et al* 2007). However, if the external motion exceeds the value observed during model building, a linear function is used to avoid large errors due to quadratic extrapolation. During the training phase, linear as well as dual quadratic models are fitted in each direction of motion. The model with the smallest DoF-adjusted error is selected. As a result, the selected model may be linear in some directions of motion and quadratic in others. Because several external signals are used, the information from the different external markers can also be weighted using the Partial Least Square (PLS) method, thus eliminating latent variables that do not contribute to the accuracy of the model (Malinowski *et al* 2012). Malinowski *et al* (2013) also investigated the effect of model updates on targeting accuracy using two statistical metrics based on the external signal alone which resulted in a similar accuracy as updates based on estimation errors but required fewer updates.

Poels *et al* (2014) proposed a method for online model update on the Vero system where newly acquired data points are used to replace old training data points at the same breathing phase (determined by linear interpolation between exhale peaks). The accuracy improvement was significant albeit very small between the clinical and online update strategies, however, the treatment time can be reduced by about 5 min on average with the online update strategy compared to the clinical update which requires treatment interruption to rebuild the model.

Poels *et al* (2015) found similar performances for the CyberKnife^®^ dual quadratic (CKDQ), CyberKnife^®^ linear and the Vero ECM on a same dataset from 15 liver and lung patients but due to the complexity of the model, the latency of internal tumour motion estimation was 15 ms for the CKDQ compared to 2 ms for the Vero model.

#### 2.3.7. Future developments in hybrid motion monitoring and motion modelling

Schnarr *et al* (2018) proposed to add a gantry-mounted kV imaging system perpendicular to the treatment beam on the tomotherapy system (Accuray Inc.) to allow hybrid motion monitoring using external optical monitoring combined with sequential monoscopic imaging.

Future software developments in hybrid motion monitoring include a 6D internal–external correlation (6D-IEC) framework using monoscopic kV-imaging in a similar workflow as COSMIK for 6 DoF hybrid monitoring (Nguyen *et al* 2018).

Going one step further, one may want to monitor the motion of the entire anatomical region including nearby OAR which may move differently from the target. Deformable motion models allow to estimate the respiratory motion of the local 3D anatomy from limited surrogate data that can be acquired during treatment (McClelland *et al* 2013). The surrogate data is often one or more external breathing signals (see section 2.1) and the model is similar to an ECM, but can estimate the full deformable motion of the local 3D anatomy. Methods have also been proposed that indirectly model the relationship between the internal motion and the surrogate data, enabling the use of real-time 2D imaging as surrogate data, such as kV-MV projection images (Vandemeulebroucke *et al* 2009) or 2D cine MR images (Stemkens *et al* 2016) (see section 2.5). Such models have been very popular in the research literature over the last 10–15 years (McClelland *et al* 2013, Thomas *et al* 2014, Stemkens *et al* 2016, Meschini *et al* 2017, Wolfelschneider *et al* 2017), but to date have seen very limited clinical use for two main reasons. Firstly, most methods require good quality 3D images which accurately depict the respiratory motion in order to build the motion models. The majority of methods proposed in the literature use 4DCT images for this purpose, however, 4DCT images only represent a single breath-cycle and so cannot be used to accurately model variability in the breathing motion. Furthermore, 4DCT images often contain sorting artefacts due to variable motion during acquisition which cause inaccuracies and uncertainties in the motion models. Recently, methods have been proposed that build the models from 4DMR datasets representing the 3D motion over several breath-cycles and including breath-to-breath variability (Stemkens *et al* 2016). One drawback is that such datasets can take a long time to acquire and process (Von Siebenthal *et al* 2007). Alternatively, methods have been proposed that fit the motion models directly to unsorted partial or raw imaging data, e.g. cine CT volumes, CT/MR slices (McClelland *et al* 2017), or CBCT projections (Martin *et al* 2014). Although promising, these methods still require further development and validation before they are suitable for clinical use. The second issue that has so far prevented the clinical adoption of deformable motion models is the lack of methods to verify and update the motion models during treatment. One of the key features of the hybrid methods is the ability to intermittently verify and update ECMs against new imaging data during treatment (section 2.3.6). However, this is more challenging for deformable motion models, since it is not possible to obtain intermittent measurements of the full 3D motion during treatment. Future research will need to focus on developing methods that use intrafractional imaging data (e.g. 2D MR) to verify and update the models and to be sufficiently confident in the accuracy of the motion estimates.

### 2.4. Add-ons to standard equipment

**Figure 5.**
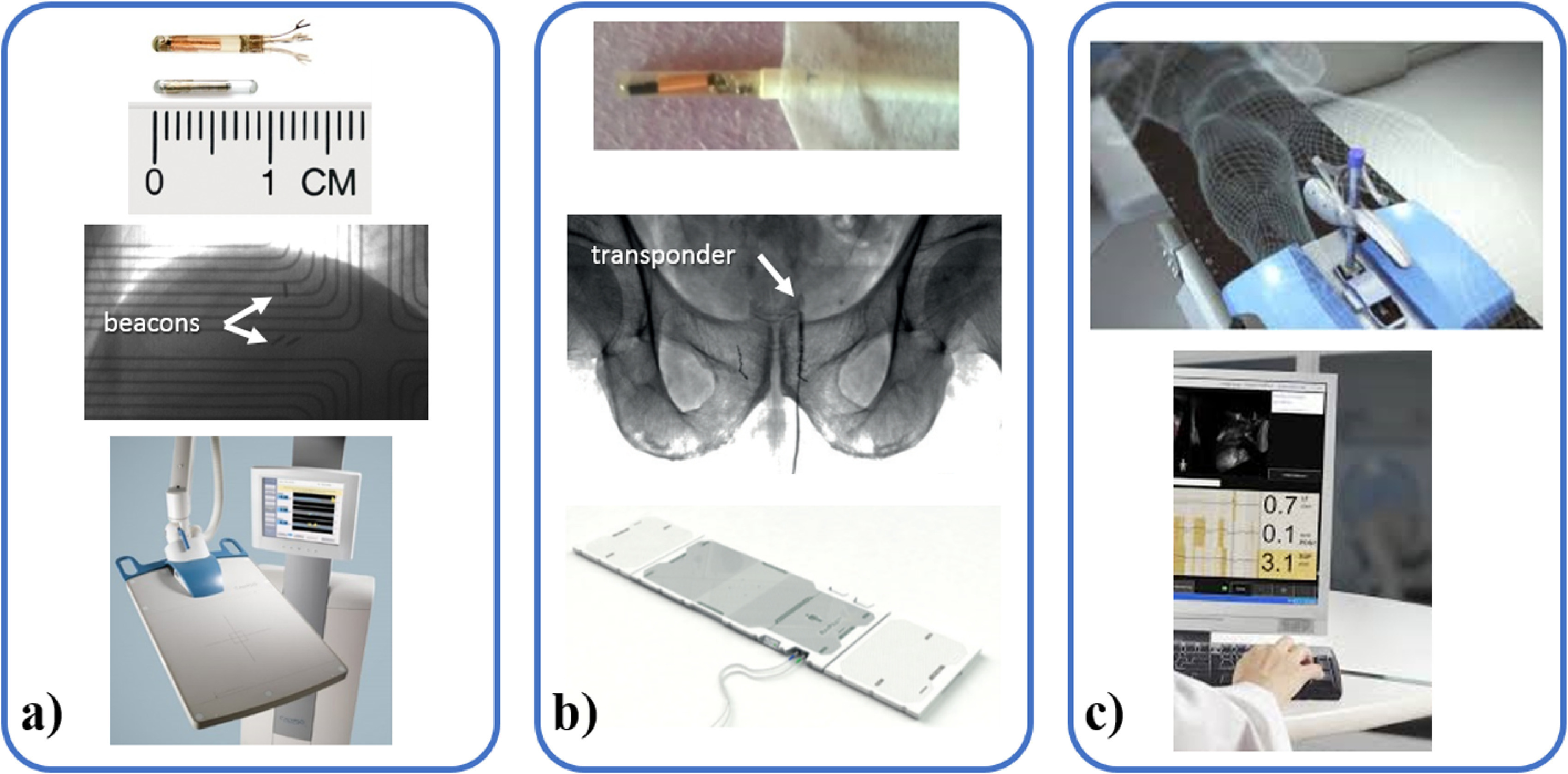
(a) An anchored electromagnetic transponder (Calypso, Varian Medical Systems) (top) can be implanted transbronchially in the lungs while 17G beacons (top, below the anchored beacon) can be implanted in any soft tissue (middle). The system is completed by an in-room antenna and console (bottom). (image provided courtesy of Varian) (b) RayPilot wired electromagnetic transponders (here shown uncoated, courtesy: Thomas Ravkilde) (top) can be implanted and removed from the prostate (middle) and plugged in a special couch (bottom) (RayPilot, Micropos Medical, Sweden). (c) The Clarity Autoscan probe (Elekta) (top) and console (bottom)(image courtesy of Elekta).

Conventional linacs can be supplemented with add-on systems for motion monitoring. Respiratory and surface monitoring were discussed in section 2.1. SyncTraX and ExacTrac were discussed in sections 2.2.2 and 2.3. Here we discuss electromagnetic transponders (section 2.4.1, figures 5(a) and (b)), and ultrasound (section 2.4.2, figure 5(d)). Note that motion monitoring using a radioactive implant (De Kruijf *et al* 2013) or emission guided radiotherapy (EGRT) based on positron emission tomography (PET) tracer detection (Fan *et al* 2012) have also been proposed. However, neither method is commercially available.

#### 2.4.1. Electromagnetic transponders/transmitters

Electromagnetic systems provide continuous real-time 3D localization of implanted transponders or transmitters without the use of ionizing radiation. The most commonly used system is Calypso (Varian Medical Systems), where the transponders are electromagnetic resonance circuits in sealed glass capsules (Balter *et al* 2005). Typically, three transponders with different resonance frequencies (300–500 kHz) are implanted in or near the treatment target. An array of excitation coils in a panel above the patient excites one transponder at a time while a second array of receiver coils localizes the resonating transponder by triangulation. It gives the 3D position of the transponder centroid relative to the panel with a frequency of 10–25 Hz. The position relative to the accelerator isocenter is determined by three room-mounted cameras that track IR markers on the panel. Although the antenna panel causes changes in beam depth dose curves and beam attenuation, its dosimetric impact on clinical treatment plans was reported to be insignificant (Zou *et al* 2013).

Calypso was first used clinically in the prostate (Willoughby *et al* 2006b), where the ability of continuous monitoring without ionizing radiation has allowed systematic investigation of motion patterns (Kupelian *et al* 2007). Studies have revealed trends like strong cranial and anterior prostate motion correlation, increased likelihood of small to medium (>3–5 mm) prostate displacements with time (but not of large displacements (>7–10 mm))(Langen *et al* 2008, Su *et al* 2011), as well as larger respiration induced prostate motion in prone position compared to supine position (Shah *et al* 2011, Butler *et al* 2013). Other clinical sites include the prostate bed following prostatectomy (Zhu *et al* 2013), pancreas (Shinohara *et al* 2012), and liver (Poulsen *et al* 2015b, James *et al* 2016, Worm *et al* 2018). In lung tissue, the stability of the smooth transponder is a challenge (Shah *et al* 2013) and an anchored version of the transponder with better attachment in the bronchia by five nitinol legs has been developed (Booth *et al* 2016, Schmitt *et al* 2017).

Drawbacks of the Calypso system include the requirement of a dedicated non-conducting couch top, lack of flexibility to move the installation between treatment rooms, a limited transponder detection volume extending maximum 21 cm below the antenna panel, and MR artifacts caused by the transponders (Zhu *et al* 2009). With a diameter of 1.85 mm (14 gauge implantation needle) the first generation of Calypso transponders were considerably larger than typical FMs, but a thinner transponder for a 17 gauge needle is now available.

A similar system is RayPilot, which consists of an implantable wired radiofrequency transmitter that receives power through a wire from a couch top plate (Kindblom *et al* 2009, Vanhanen and Kapanen 2016). The couch top plate houses receiving antennas that detect the transmitter position and orientation at 30 Hz. The transmitter is implanted transperineally in the prostate with the wire passing through the perineum of the patient, and it is removed after treatment completion. Recent clinical studies found that the implantation and explantation procedures were feasible and safe, but the studies also reported interfractional transmitter position instabilities and recommended to combine real-time prostate motion monitoring by RayPilot with an independent IGRT system for daily prostate localization (Braide *et al* 2018, Vanhanen *et al* 2018). A newer version of the RayPilot, HypoCath, is catheter-based to remove the need for surgical intervention and allows to localize the urethra as well as the prostate.

#### 2.4.2. Ultrasound methods

Ultrasound (US) systems are capable of continuous image acquisition in real-time with good soft tissue contrast, while not exposing the patient to additional ionising radiation. This enables direct monitoring of internal tissue motion and deformation at high spatial and temporal resolutions. Clarity Autoscan^™^ (Elekta) (figure 5(c)) is currently the only commercial US system designed for intrafraction motion monitoring. Approved specifically for prostate and prostate bed radiotherapy, the system incorporates a 3D transperineal US (TPUS) probe and is compatible with standard C-arm linacs. As such, Autoscan provides a flexible, cost-effective monitoring system that is unaffected by metal hip prostheses and does not require implanted FM. Integration with Elekta linacs enables motion mitigation via automated couch correction or gating, typically at an action threshold of 3 mm for 5 s, which can be varied if desired.

The US probe comprises a mechanically swept curvilinear transducer array with a 5 MHz centre frequency, which is secured to a baseplate to hold it in place during treatment. Sweeping the transducer array produces a continuously scanned 3D field of view. During monitoring, template matching based upon normalized cross correlation is used to automatically estimate the motion of a target reference volume within the imaging field of view (Lachaine and Falco 2013). The reference volume position is encoded in room coordinates by optically monitoring IR markers on the Autoscan probe using a room mounted stereoscopic camera (Polaris Spectra, NDI, Canada). Monitoring rates of ~0.5 Hz are employed for prostate motion monitoring.

Autoscan’s accuracy was validated *in vivo* against manual localization of intraprostatic markers in EPID images (Grimwood *et al* 2018, Han *et al* 2018) and against RayPilot monitoring (Delcoudert *et al* 2017). Characterisations of prostate motion during treatment describe a gradual drift from the isocentre with substantial inter-patients variations showing maximum recorded shifts  >10 mm and a mean SI drift of 0.075 mm min^−1^ (Ballhausen *et al* 2015, Li *et al* 2017).

As a soft tissue imaging modality, US is able to monitor a range of anatomical surrogates where the lesion cannot be discerned. This has motivated the use of experimental ultrasound systems to study a range of treatment sites beyond the prostate. The upper abdomen is of particular interest, because it is susceptible to respiratory motion and is largely accessible to US without obstruction from bony anatomy.

Liver motion monitoring using an adapted Vivid 7 Dimension probe (GE Healthcare, USA) was evaluated against Calypso in a free-breathing patient immediately after liver SBRT (Ipsen *et al* 2017). Another group has pioneered the use of an experimental version of Clarity to monitor the 3D position of the liver in 13 patients during RT delivered in breath hold (Boda-Heggemann *et al* 2016, Sihono *et al* 2017, Vogel *et al* 2018). A 3D US probe was held using a mechanical arm against the rib-cage throughout planning CT, CBCT and RT delivery, without interfering with treatment delivery (Boda-Heggemann *et al* 2016). The residual intra-breath-hold motion (e.g. drift) measured using US during CBCT acquisition was found to correlate well with residual motion measured from CBCT projection images (Vogel *et al* 2018).

US was also used for motion monitoring of the pancreatic head and surrogate structures, including the superior mesenteric artery and portal vein (Omari *et al* 2016) as well as for diaphragm position monitoring as a surrogate for lung tumour position (Mostafaei *et al* 2018).

US has been combined with MLC tracking *in vitro* (Fast *et al* 2016, Ipsen *et al* 2016) with a total system latency of ~1 s, therefore demonstrating adequate compensation for the slow motion typically observed in prostate cancer (Fast *et al* 2016, Colvill *et al* 2014). A predictive compensation method was demonstrated on sinusoidal target movements, reducing system latencies to 172 ms (Ipsen *et al* 2016). This technique illustrates a potential approach to compensate for monitoring latency of breathing motion in lung radiotherapy patients, but requires further *in vivo* evaluation.

Despite the scarcity of clinical free breathing patient studies, promising findings have also arisen from the MICCAI Challenge on Ultrasound Liver Tracking (CLUST), which comprises an open dataset of labelled anatomical features in 64 2D and 22 4D *in vivo* image sequences (De Luca *et al* 2018). Using results from CLUST, an estimation of the impact from monitoring on treatment margins was made, indicating a possible 75% reduction.

Optimal imaging requires careful probe placement to maximise patient-probe contact and to ensure adequate anatomical coverage in the field of view. Fargier-Voiron *et al* (2016) and Li *et al* (2017) have identified a need to control for anatomical deformation and changes to image quality associated with variations in probe pressure. Furthermore, at patient set up, the probe must be manually adjusted to ensure both reproducible positioning and adequate target volume coverage. Approaches to assist with probe-positioning are being investigated (Camps *et al* 2017, 2018). Remote probe support and robotic systems are also being developed to optimise probe placement during both patient set up and treatment delivery (Schlosser *et al* 2012, Sen *et al* 2017, Su *et al* 2017a). The implications of placing an ultrasound probe within the gantry arc require further consideration of the resulting beam attenuation. Monte Carlo probe models have been developed for incorporation with planning software (Bazalova-Carter *et al* 2015) and the integration of robotic ultrasound with the CyberKnife^®^ system has also been examined (Gerlach *et al* 2017). Another mitigation strategy has been pursued whereby a probe was manufactured using radiolucent materials to reduce interference with the treatment beam (Schlosser and Hristov 2016). Finally, an autonomous system for avoiding the treatment beam altogether has also been demonstrated (Schlosser *et al* 2016).

### 2.5. Magnetic resonance imaging

Recently, radiotherapy machines with integrated MR imaging have entered clinical practice (Paganelli *et al* 2018). There are currently two commercially available MR-guided treatment systems: the ViewRay MRIdian and the Elekta Unity system (figure 2(d)) (Raaymakers *et al* 2009, Lagendijk *et al* 2014, Mutic and Dempsey 2014, Mutic *et al* 2016). Additionally, two research groups operate prototype systems (Fallone 2014, Keall *et al* 2014a, Liney *et al* 2016). The prospect of monitoring intrafractional anatomical changes and guiding real-time adaptive radiotherapy with MR imaging was one of the driving forces behind the development of these machines. MR imaging offers excellent soft-tissue contrast and does not require FM implantation or expose the patient to additional imaging dose. However, cancer patients with metal implants (e.g. prostetics, pacemakers) or very large patients cannot be examined using MR imaging.

**Figure 6.**
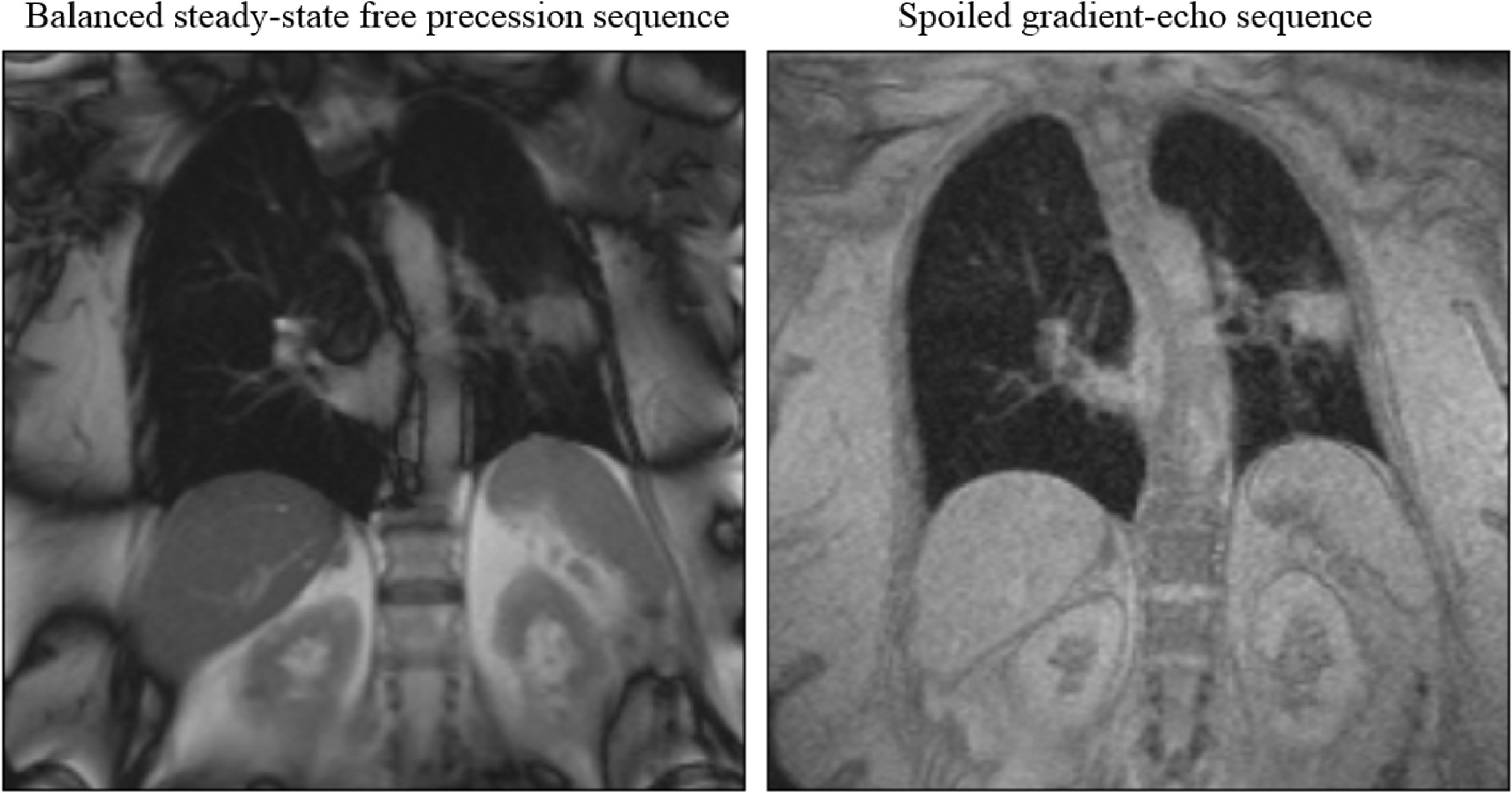
Two coronal 2D cine MR images of a lung cancer patient acquired with different gradient-echo MR sequences. One has been acquired with a balanced steady-state free precession sequence providing a T2/T1-weighted contrast, while the other was obtained using a spoiled gradient-echo sequence with a T1-weighted contrast (Menten *et al*, unpublished).

It is not yet possible to acquire, reconstruct and postprocess 3D MR images at an adequate resolution and imaging rate to monitor fast motion. Instead, 2D cine MR imaging, which is able to survey one or multiple 2D imaging planes in real-time, may be harnessed to monitor fast-moving tumors and OAR. Pioneered in cardiac imaging, cine MR imaging is usually based on gradient-echo MR sequences deploying a single radiofrequency pulse (Bernstein *et al* 2004). This sequence design permits the use of very short echo times and, consequently, shorter repetition times, resulting in sub-second acquisitions. Varying these settings as well as adding additional sequence components, such as preparation pulses, allows measurement of different image contrasts (figure 6). In addition to different contrasts, the image resolution, position and orientation may be adjusted. It is also possible to successively survey multiple imaging planes in order to acquire some volumetric information. All these imaging parameters influence the maximum imaging rate, typically in the order of a few images per second. Additionally, scanner specifications, such as strength of the main and gradient magnetic field and read-out electronics, impact the achievable contrast and acquisition speed.

Image acquisition can be further accelerated by reducing the amount of acquired k-space data. This results in either a lower image resolution or a smaller field-of-view. Should neither be acceptable, parallel imaging techniques can be deployed to reconstruct undersampled k-space data using multiple independent coils to record the subject’s MR signal (Deshmane *et al* 2012). As the signal measured by each coil depends on its position relative to the patient, this additional spatial information can be used during image reconstruction. It should be noted that the parallel imaging capabilities of most MR-linacs are still limited. While diagnostic MR scanners with 32 or more individual coil channels are commercially available, equivalent hardware is still lacking for MR-guided radiotherapy systems.

2D cine MR imaging can be used to either determine the tumor position directly or indirectly by locating a surrogate structure whose movement is correlated with the target motion. In the future, it may also be used to monitor target deformations and rotations as well as track nearby OAR. Deformable motion models could also be used to estimate the local 3D anatomical motion from 2D cine MR images, as discussed in section 2.3.7 (Stemkens *et al* 2016, Tran *et al* 2018). Multiple algorithms have been designed to accurately, reliably and quickly extract the position or outline of a volume-of-interest from 2D cine MR images (Cerviño *et al* 2011, Shi *et al* 2014, Mazur *et al* 2015, Paganelli *et al* 2015, Yun *et al* 2015, Feng *et al* 2016, Bourque *et al* 2016, Yip *et al* 2018) with an accuracy approaching inter-observer variability.

So far, most algorithms rely on a set of training contours from 2D cine MR images of the same patient. In a clinical workflow, the training data can be collected as part of pre-treatment imaging and reliable manual contours can be created while the patient is being prepared for treatment. Potentially, algorithms trained on an independent cohort of patients could be used. Several papers have presented promising segmentation tools for 3D biomedical images based on deep learning (Ronneberger *et al* 2015). However, this has not been explored yet for 2D cine MR imaging in a radiotherapy context. Currently, obtaining a training dataset of sufficient size proves difficult as 2D cine MR images are rarely acquired in clinical routine.

Localization accuracy in a 2D plane does not necessarily translate into usefulness to determine an anatomical structure’s position and extent in three dimensions. The volume-of-interest may shift perpendicularly to the imaging plane or move out of it entirely. For this reason, multiple studies have seeked to optimize the number and orientation of 2D cine MR images for real-time adaptive radiotherapy (Bjerre *et al* 2013, Tryggestad *et al* 2013, Brix *et al* 2014, Ipsen *et al* 2016, Menten *et al* 2018). While most of these studies show that 2D cine MR imaging can be used to localize a volume-of-interest in three dimensions, no consensus strategy on image orientation and imaging parameters can be derived from the literature. Both, the ideal imaging strategy and deployed image processing may depend on the cancer site monitored as well as the desired intrafractional adaptation strategy.

MR guidance for intrafractional motion monitoring is still at its beginning. However, few clinics have begun to deploy on-board MR imaging to guide intrafractional treatment beam gating on the ViewRay MRIdian (Green *et al* 2018, Henke *et al* 2018, Tetar *et al* 2018). Gating with an average system latency of 394 ms is based on a single sagittal 2D cine MR image acquired using a balanced steady-state free precision sequence at four frames per second. At Washington University, St. Louis, MO, USA, site of the first MR-guided treatment, approximately one third of patients undergoing MR-guided radiotherapy are treated with gating (Fischer-Valuck *et al* 2017) mostly for the treatment of thoracic and abdominal tumors. Results from initial clinical trials (Acharya *et al* 2016, Henke *et al* 2018) and further research studies will provide much needed experience about the potential of MR imaging for intrafractional motion monitoring.

## 3. Validation and QA

### 3.1. Validation tools for development and early implementation

A small number of studies have used animals for motion monitoring end-to-end testing (Shchory *et al* 2009, Poulsen *et al* 2012a). While animal experiments represent a realistic end-to-end test, they are difficult to perform and may pose ethical concerns. In addition, ground truth motion is unknown in animal subjects and experiments are not reproducible. End-to-end experiments using commercially available moving phantoms allow reproducible testing of the technical components as well as to evaluate the accuracy and the latency of intrafraction motion monitoring but they lack the realism of human subjects in terms of image quality or complexity of motion.

Malinowski *et al* (2007) proposed a motorized platform which can be used to move a rigid phantom or dosimeter with high reproducibility (table 3). Anthropomorphic phantoms which provide a more realistic representation of patient anatomy during end-to-end tests were also developed (Biederer *et al* 2006, Nioutsikou *et al* 2006, Kashani *et al* 2007, Remmert *et al* 2007, Serban *et al* 2008, Steidl *et al* 2012, Haas *et al* 2014, Cheung and Sawant 2015, Perrin *et al* 2017) with some representative examples summarized in table 3. The representation of ribs is particularly important in thoracic particle therapy since the presence (or absence) of a rib on the particle beam path may result in under (or over-) shoot of the particle beam’s Bragg peak. Detailed features such as vasculature and airways are important for accurate deformable image registration in motion modelling. There is typically a trade-off between realism/anthropomorphism and motion trajectory reproducibility and the use of animal tissue requires careful expert manipulation and controlled laboratory conditions (Biederer and Heller 2003). Highly realistic phantoms can also be generated using 3D printing technology although this has been limited to static versions so far (Hazelaar *et al* 2018b).

**Table 3.** Physical phantoms developed by research group.

Phantom (site)	Deformable/anthropomorphic	Motion reproducibility	Main features
WashU (any site) (Malinowski *et al* 2007)	No/no	• Target accuracy (mean ± SD) < 1 mm	• 3D axis and independent 1D vertical axis
			• Motorized platform to carry phantom or dosimetry equipment

LuCa (lung) (Perrin *et al* 2017)	Yes (interior and exterior)/yes (high level of detail)	• Stable end in/exhale (<1 mm)	• Inflatable/deformable lungs, skeleton, muscles skin, solid heart, solid mobile tumour (can hold dosimetric films)
		• Tumour position varied from day to day for a given intermediary in/exhale pressure	• Motion actuated by an air pump inflating the lungs
			• MR-compatible with visible deforming lung features

Lung (Cheung and Sawant 2015)	Yes (interior and exterior)/yes (low level of internal detail)	• <2 mm day-to-day (ascribed to set-up)	• Deformable external shell
			• Latex foam insert for lungs
		• < 0.25 mm RMS intra-day	• Rigid foam diaphragm actuated by the WashU motion stage

Lung (Biederer *et al* 2006, Remmert *et al* 2007)	Yes (interior)/yes (animal heart and lungs, nodules, airways, no ribs)	• Maximal diaphragm displacement precision (SD) 1.90 mm (on CT), 1.47 mm (on MR)	• Porcine lung and heart explants with tracheal tube in saline solution, artificial pulmonary nodules
			• Water-filled silicon diaphragm inflated or deflated by a water pump outside the MR room
		• Reproducibility of intermediary phases not quantified	• MR-compatible

Lung (Serban *et al* 2008)	Yes (interior)/yes (only one lung with vasculature/airways features)	• Within image resolution (0.7 × 0.7 × 1.25 mm^3^)	• Lung (natural latex balloon filled with damp sponges) in water, thoracic cavity (Lucite), diaphragm (motor-actuated piston), tumour (Dermasol ellipsoid), vascular and bronchial bifurcation (nylon wires and Lucite beads)

Lung (Steidl *et al* 2012)	Yes (exterior)/yes (low level of internal detail, cubic tumour)	Target accuracy (mean ± SD) = 0 ± 0.09 mm (input versus log files)	• Artificial skeleton, rubber skin
			• Tumour: PMMA cube with 20 slots for pinpoint ion chambers and 5 films
			• Sternum-induced thoracic motion
			• 6D robot-actuated tumour motion independent of thoracic motion

MAESTRO (lung) (Haas *et al* 2014)	Yes (ribcage only)/yes (no vasculature)	• Millimetre positioning precision	• Mechanically actuated ribs, stationary lungs, trachea and spine in hermetic skin (to be filled with water)
		• Inter-cycle reproducibility <0.16 mm RMS	• Robot-actuated tumour motion

ELPHA (liver) (Ehrbar *et al* 2019)	Yes (interior)/yes (liver with vasculature)	• Reproducibility <0.32 mm RMS (inter- and intra-day)	• Soft silicon liver with vasculature (can hold dosimetric devices)
			• Static inferior plate and motor-driven superior plate
			• Ultrasound and CT contrast

Computer simulations are also an important part of validation for two main reasons: first, experiments are time-consuming and simulations allow a larger data–set to be obtained providing better statistics in a shorter time. Second, simulations allow comparison of various methods with perfect reproducibility as well as exploration of other hardware configurations not necessarily available to the user (Cho *et al* 2012, Bertholet *et al* 2018, Montanaro *et al* 2018). Digital phantoms may be particularly useful for simulations involving multi-modality imaging (Segars *et al* 2010, Mishra *et al* 2012, Paganelli *et al* 2017). The XCAT phantom was based on visible male and female anatomical datasets from the National Library of Medicine (Segars *et al* 2010, National Library of Medicine). The heart motion model was derived from high resolution cardiac-gated multi-slice CT angiogram. The breathing motion model was derived from respiratory gated-CT of healthy subjects and is controlled by chest and diaphragm motion curves. The phantom has allowed other researchers to closely reproduce tumour shape and location and motion seen in patients (Mishra *et al* 2012) and to adapt it for MR imaging with detailed imaging parameters (Paganelli *et al* 2017). While state-of-the-art digital phantoms can simulate realistic looking motion and images and are a valuable tool for validation, it is not known how accurately the simulations represent the real motion that can occur in human subjects, and they do not enable the end-to-end testing that can be performed with hardware phantoms.

Motion traces used for simulations and experiments should also be carefully chosen. Site-specific motion traces measured in patients should be used in generaly and internal traces should be preferred to inferred traces especially for the validation of hybrid monitoring methods relying on internal–external correlation or monoscopic imaging methods relying on inter-dimensional correlation (Montanaro *et al* 2018).

Note that marker/tumour segmentation errors or uncertainties cannot be reproduced without patient data and have to be assessed independently in retrospective clinical studies.

### 3.2. Quality assurance

An important limiting factor for the implementation of motion monitoring in clinical practice is the lack of sufficient QA procedures. Especially in combination with real-time adaption (tracking) where a treatment plan validated pre-treatment is modified on the fly, standard patient-specific QA procedures are no longer sufficient. The critical review by De Los Santos *et al* (2013) and references herein discuss the QA procedures specific to different motion monitoring and/or real-time adaptation equipment. The AAPM TG-135 provides recommendations for QA of robotic radiosurgery (Dieterich *et al* 2011), AAPM TG-154 provides recommendations on in-room US QA (Molloy *et al* 2011), AAPM TG-104 provides recommendations for non-radiographic localization systems such as external and electromagnetic methods (Willoughby *et al* 2012). For methods using linac mounted kV and MV imaging, the regular linac commissioning methods described by AAPM TG-104 and AAPM TG-142 cover geometrical and image quality QA (Fang-Fang and John 2009, Klein *et al* 2009). To complete the QA program for KIM, Ng *et al* (2014) proposed additional tests based on the existing QA program for the Calypso system (Santanam *et al* 2009). These tests included verification of the static localization accuracy, the dynamic localization accuracy, the treatment interruption accuracy, latency measurements and clinical conditions accuracy.

Important considerations for QA procedures are the latencies and geometric tolerances as well as the complexicity and frequency of the tests. Ng *et al* (2014) chose a 1 mm geometric tolerance for the KIM QA program as it is well below typical margins and in line with other geometric errors such as isocenters or couch calibration. In order to set-up a program that is both efficient and effective, Sawant *et al* (2010) used the failure mode and effect analysis (FMEA) framework to determine the frequency of QA tests for Calypso-guided MLC tracking. The industrial engineering FMEA framework consists of (i) charting a process tree identifying each step of the procedure (in this case: motion monitoring and adaptation), (ii) identifying the potential failure modes at each step, (iii) identifying the corresponding potential causes and their downstream effects and, (iv) quantifying the overall risk of the failure based on the probability of occurrence (O), severity of the effect (S) and detectability (D). O, S and D scores (from 1 to 10) can be multiplied to obtain the overall risk probability number (RPN). RPN scores were obtained from a group of MLC tracking experts and tests for failure modes with a score above 125 were recommended to be performed monthly while other failure modes were recommended as part of commissioning, annual QA and after major hardware/software upgrades. The resulting MLC tracking-specific QA program adds ~35 min to monthly QA and ~3.5 h for comprehensive testing.

For MR-linacs, interactions and interfacing of monitoring and treatment delivery tests have to be performed in addition to conventional MR scanner and linac QA tests (Tijssen *et al* 2019). Hybrid tests were therefore tailored to the RT-specific aspect of MR imaging and the hardware modifications necessary to integrate the two modalities. In particular requirements for geometric fidelity on a large field of view are stricter for MR-guided RT than for diagnostic MRI (Ginn *et al* 2017, Tijssen *et al* 2019). All QA tests need to be performed with MR-safe and/or compatible equipment.

## 4. Translation to particle therapy

The translation of photon therapy motion monitoring concepts to particle therapy facilities was mentioned in numerous publications (Riboldi *et al* 2012, Shirato *et al* 2012, Seco and Spadea 2015, Knopf *et al* 2016, Kubiak 2016, Trnková *et al* 2018). However, only few studies have shown results from such translations (Shimizu *et al* 2014, Umezawa *et al* 2015, Mori *et al* 2016). Efforts to translate motion monitoring and motion mitigation approaches are challenged by stricter accuracy requirements in particle therapy than in photon therapy. Particle dose distributions have a steeper dose fall-off at the distal edge of the Bragg peak and are sensitive to inline anatomical changes. Furthermore, in particle beam scanning (PBS), the interplay effect challenges the dose homogeneity for moving targets. As a result, millimetre uncertainties can result in significant target dose miss or OAR overdosage.

**Figure 7.**
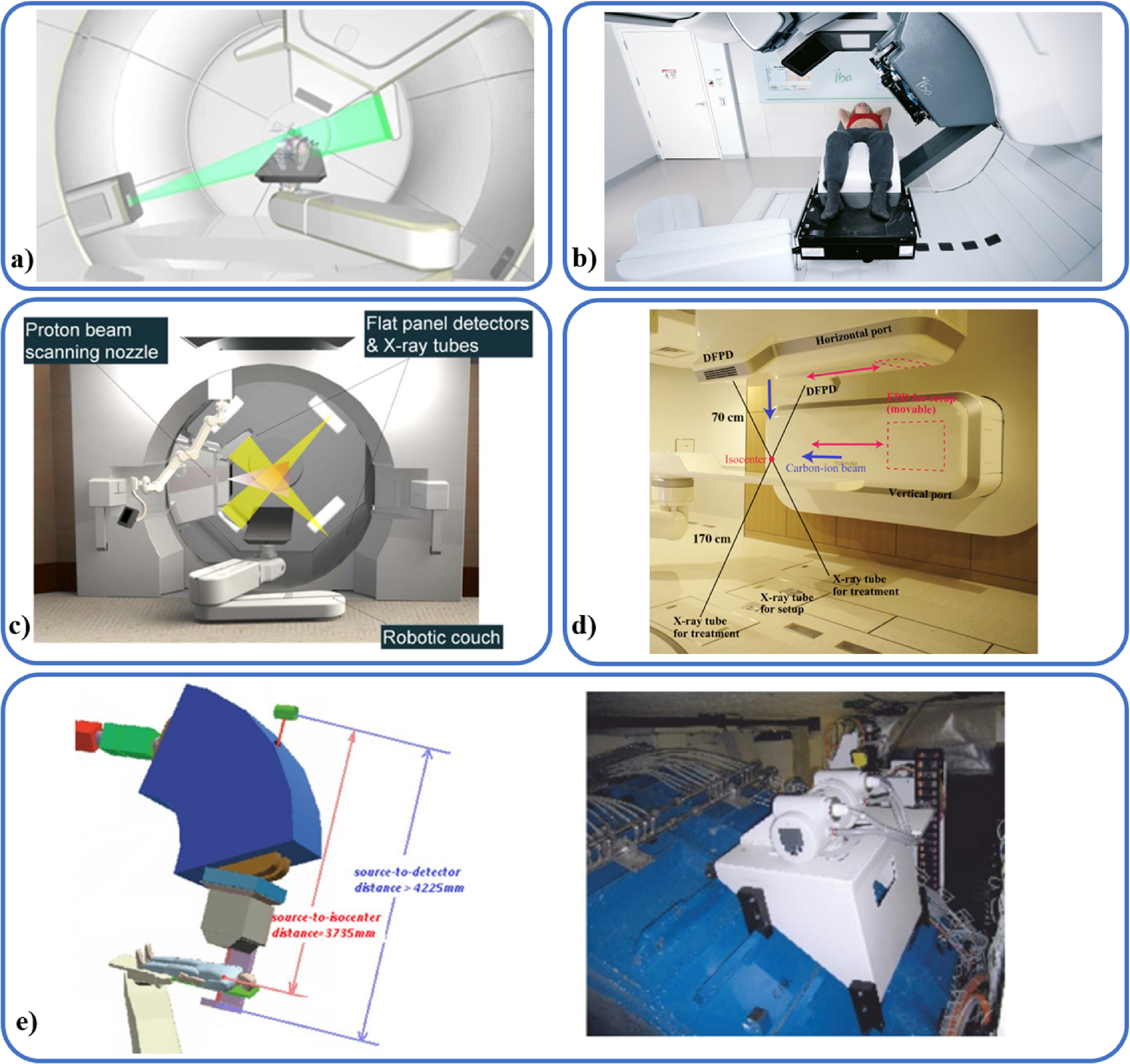
Imaging during particle therapy. Gantry mounted CBCT systems on (a) the Varian probeam (Image provided courtesy of Varian) or (b) the IBA system could be used in real-time fluoroscopy mode. Stereoscopic imaging was integrated with (c) proton beam scanning (reprinted from Shimizu *et al* (2014) under CC BY licence) and (d) carbon ion beam scanning (reprinted with permission from Mori *et al* (2016)). (e) BEV x-ray imaging is available only at the PSI facility. The photo shows the x-ray tube mounted on the final bending magnet (reprinted with permission from Zhang *et al* (2013)).

Particle therapy facilities are nowadays equipped with similar in-room imaging capabilities as photon therapy facilities (figure 7). For patient positioning, orthogonal kV imaging was available early-on (figures 7(a) and (b)) and can potentially be used in fluoroscopy mode to track the movement of anatomical structures or markers as suggested for the real-time-image gated, spot-scanning proton beam therapy (RGPT) system at the Hokkaido University (figure 7(c)) (Shimizu *et al* 2014, Umezawa *et al* 2015) or for carbon-ion scanning (figure 7(d)) (Mori *et al* 2016). A specific x-ray imaging implementation is available at the Paul Scherer Institute (PSI), enabling BEV imaging (figure 7(e)) (Pedroni 2006, Safai *et al* 2012). Zhang *et al* (2013) describe a method by which 3D motion can be extracted from such a monoscopic, real-time imaging system. Optical surface imaging was introduced in proton therapy facilities over the last years (Batin *et al* 2016) and showed to be more robust in monitoring respiratory motion than electromagnetic monitoring in controlled laboratory conditions (Fattori *et al* 2017). Furthermore, efforts are made towards hybrid motion monitoring system (Cho *et al* 2017) using optical systems in combination with fluoroscopy systems. Optical imaging may have a more important role to play in monitoring patient motion during particle therapy and respiratory motion management than pre-treatment patient positioning when compared to volumetric CBCT/in-room CT image guidance methods (Ciocca *et al* 2016, Fattori *et al* 2016). Clinical application of ultrasound imaging in particle therapy has been rare, yet a phantom-based experiment has shown that real-time ultrasound motion detection and beam tracking enable considerably reduced interplay effects in scanned ion beam radiotherapy (Prall *et al* 2014).

Also, more and more studies about online MR-guided proton therapy have been published in the recent years (Raaymakers *et al* 2008, Wolf and Bortfeld 2012, Moteabbed *et al* 2014, Hartman *et al* 2015, Oborn *et al* 2015, Fuchs *et al* 2017, Schellhammer *et al* 2018), envisioning new ways to enable motion monitoring and mitigation. A recent review paper by Oborn *et al* (2017) predicted the accelerated development of hardware and simple prototype systems within a few years and coupled systems integrated with gantries in a decade. For the time being, online MR-guided proton therapy however remains a pure research topic far away from clinical implementation.

Despite the availability of imaging equipment, the provided information is often not sufficient to employ the same motion monitoring and motion mitigation concepts as for photon therapy. Surrogate motion information (e.g. from an implanted marker) might not be sufficient in particle therapy to guarantee target dose coverage. This is due to the sensitivity of particles not only to geometrical changes but also density changes along the beam path. Thus, to accurately assess the influence of motion on particle dose distributions, 4D anatomical images of the whole patient geometry within the beam path are required. Currently, mainly static targets are treated at proton therapy facilities. If at all, moving targets are treated in breath-hold or with gating (Minohara *et al* 2000, Bert *et al* 2009, He *et al* 2014, Zhang *et al* 2015, Yamada *et al* 2016). Tracking by steering the proton beam according to the target motion remains a research topic (Bert *et al* 2007, Grözinger *et al* 2008, Zhang *et al* 2014b).

Implanted FMs are associated with specific particle therapy-related challenges requiring particular precaution (Kubiak 2016). Although commercially available markers are popular in photon radiotherapy, the feasibility of their direct implementation in particle therapy is still under investigation. In the PROMETHEUS trial carried out at the Heidelberg Ion Beam Therapy (HIT) Center, different markers were evaluated for suitability for the treatment of hepatocellular carcinoma using scanned ion beams (Habermehl *et al* 2013). A concern for the use of FMs in particle therapy is that they are made of high-Z materials causing unfavourable artefacts in conventional CT scans (Schlosser *et al* 2010). The inaccurate representation of the electron density and thus Hounsfield units near the inserted clips may result in improper dose calculation (Habermehl *et al* 2013). Furthermore, metal markers can interact with particle beams (particularly scanned ion beams) and have a considerable impact on the therapy (Bert and Durante 2011). The degree of their influence on the dose distribution, fluence and range of ions depends on the material, thickness and location in the treatment field. Only thin markers (<0.5 mm) or those made of relatively low-*Z* materials, e.g. carbon-coated zirconium oxide clips, may be considered for use in particle therapy (Habermehl *et al* 2013). Electromagnetic localization of internal transponders is an alternative method of motion detection. At PSI the TULOC system was developed and successfully tested (Seiler *et al* 2000) although it has not been used clinically. An alternative implementation is the Calypso system described in section 2.4.1 (Balter *et al* 2005). In their review, Landry and Hua (2018) point out that electromagnetic monitoring systems currently suffer from significant distortions which limit their use in a clinical particle therapy.

Precise motion monitoring is the premise for adaptive 4D particle therapy. Most publications agree that the impact of motion in particle therapy (especially PBS) is highly individual for a specific set of patient characteristics and machine parameters as well as their specific combinations per treatment fraction. Thus, it is hard to predict dosimetric consequences of the tumour motion in prospective multiple scenarios evaluations. More and more publications underline the value of log file based dose reconstruction and accumulation to move towards 4D adaptive PBS particle therapy (Klimpki *et al* 2018, Krieger *et al* 2018, Pfeiler *et al* 2018). For such approaches, high-frequency, low-latency, synchronized motion monitoring data is required. 4D-dose-accumulation treatment-assessment tools are in the phase of clinical implementation (Meijers *et al* 2019), allowing for a quality assessment of the 4D delivered dose throughout the treatment course triggering decisions for plan adaptations, in case of significant deviations.

## 5. Conclusions and outlook

This review compared and analysed the different real-time motion monitoring methods that have been clinically demonstrated. It illustrates the variety in hardware-focused methods (e.g. stereoscopic imaging, dedicated tracking machines, MR-linac) and software-focused methods on standard-equipped linacs (e.g. KIM, sequential stereo, COSMIK, kV/MV monitoring). Add-on equipment represents a middle ground albeit also covering a spectrum between out-of-the-box systems (e.g. Calypso) and more processing-intensive or user-dependant methods (e.g. ultrasound). In all three categories, effort has been made to monitor soft tissues and tumours rather than internal or external surrogates with the MR-linac as a dedicated machine, US as an add-on imaging technology and markerless monitoring of lung tumours and bronchi on conventional linacs. However x-ray imaging is limited by its inherently poorer soft tissue contrast than MR or US imaging. The choice of equipment and method(s) to implement depends on three main factors. First: the treatment site. Respiratory surrogate and hybrid monitoring for example are not applicable for prostate where gastro-intestinal activity dominates organ motion. The strong reflection of ultrasound at tissue/air interfaces makes ultrasound imaging a contraindication for direct lung tumour monitoring. Markerless x-ray based monitoring is difficult in large patients as well as in the abdomen and pelvis due to poor contrast. Second: the motion mitigation strategy. A high monitoring frequency may not be necessary for gated prostate or spine treatments because of the slow motion. However, large excursion of the prostate due to gas movement may require monitoring with a higher frequency in extreme hypofractionated prostate RT. On the other hand, tracking tumours that move with respiration requires a high-frequency low-latency signal in combination with prediction algorithms. Hybrid monitoring is well suited for respiratory gating where kV imaging can be optimally used during MV beam-on time only. Latency of motion monitoring methods are generally calculated indirectly from the entire real-time adaptation system latency. The AAPM task group 76 report suggests that the total latency period of a real-time tracking system should be kept as low as possible and below 0.5 s for respiratory motion because of prediction algorithms limitations (Keall *et al* 2006). Given the slower motion of certain targets such as the prostate or the spine, imaging rates and monitoring latencies of a second or more may be acceptable for these targets. Similarly, baseline drift correction and tumour trailing for sites affected by respiratory motion may not require a latency as low as 0.5 s. The third factor is material and human resources. A specialized machine may be optimally used in large centres where a large volume of patients justifies the investment and staff training. Smaller centres may prefer the versatility of standard-equipped linac methods or mobile add-on equipment. FM or electromagnetic transponder/transmitter implantation is also a complex procedure requiring specific radiologist/bronchoscopist training and a good coordination in scheduling between different services.

This review also points out the variety of metrics used in reporting target motion amplitude and motion monitoring accuracy. Percentile ranges are useful to determine ITV margins. Population mean and SD of motion are often reported because they directly translate to random and systematic component of margin calculation (van Herk 2004) while the RMS, also known as quadratic mean, is less frequently reported. Yet, population-based measures do not adequately represent the variety in individual motion patterns. The amount of time the target spends at a certain distance from its planned position may also be useful to determine the margin robustness to motion. Different measures are therefore pertinent to different sites and applications and can be reported on a population or on a per-patient basis. In order to facilitate the comparison of motion monitoring reports, we recommend to include population mean and SD for all directions of motion as well as the maximum mean and SD of motion observed in a single patient and fraction to illustrate outlying but nonetheless realistic cases.

The accuracy of motion monitoring methods can be reported with similar measures as target motion. BEV errors are sometimes reported instead of errors in each directions of motion. BEV errors may be sufficient for photon therapy but inline errors should also be considered in particle therapy due to range uncertainty. As mentioned in the introduction of section 2, accuracy is often defined as the mean error which is not compliant with the ISO 5725-1 standard (ISO 1994). We recommend that motion monitoring methods are described by their accuracy as the combination of the trueness (error mean) and precision (standard deviation).

Motion mitigation is an obvious application of motion monitoring and several mitigation methods have been compared in different treatment sites (Menten *et al* 2012, Ehrbar *et al* 2016, 2017a, Colvill *et al* 2016, Toftegaard *et al* 2017, Nankali *et al* 2018). Another application of motion monitoring is real-time dose reconstruction which can provide real-time QA for treatments delivered with or without mitigation (Ravkilde *et al* 2014, 2018, Kamerling *et al* 2016a, 2017). Motion monitoring and real-time dose reconstruction are the essential foundation of online replanning (Kamerling *et al* 2016b, Kontaxis *et al* 2017). Motion-including dose reconstruction can also help to develop dose-response models and evaluate clinical outcome based on the actually delivered dose instead of the planned dose (Bentzen *et al* 2010, Siochi *et al* 2015, Meijers *et al* 2019).

IGRT—the integration of imaging and treatment in a single machine—revolutionized radiotherapy and has opened ‘many doors for exploration’ (Jaffray 2012). The exploration of x-ray based imaging resulted in the clinical implementation of many methods discussed in this review and more developments are still ahead (see sections 2.2 and 2.3). Even more doors are now open with a new form of IGRT: MR guidance. Progress in image processing and robotics may also facilitate wider implementation of US imaging. Particle therapy puts higher demands on motion monitoring than photon therapy. At modern proton facilities, almost the same imaging capabilities are nowadays available as in photon therapy. If they will be employed in the same way in clinical routine remains to be shown in the coming years.

The methods presented in this review were developed and implemented over about 20 years with increasing level of surrogate quality and dimensionality. The state-of-the art has shifted from respiratory surrogate monitoring, to single and to multiple implanted marker monitoring and ultimately, imaging the tumour itself and/or the surrounding soft tissue with MR or US imaging. In the same fashion, 1D breathing signals and 2D imaging were replaced by 3D inferred or triangulated positions and 6 DoF monitoring while multiple object monitoring and motion models are aiming at monitoring the position of the target and the surrounding organs which may move differently than the target. This evolution shows that the community not only wants to ‘see what we treat as we treat’ but wants to see it in ever more detail. There is also a growing interest in performing functional imaging during treatment (Fan *et al* 2012, Datta *et al* 2018). Functional imaging or the monitoring of biological functions such as blood flow and cellular dynamics are not yet feasible in real-time in a radiation therapy setting, and as such were considered beyond the scope of this review. However, these effects likely play an important role in tumour control and toxicity effects of radiation therapy. As well as the introduction of imaging in the treatment room (IGRT) paved the way to real-time motion monitoring of tumour and OAR position, the introduction of functional imaging in the treatment room is likely to open the way to real-time biology-guided radiation therapy.
